# Design and synthesis of propellane derivatives and oxa-bowls via ring-rearrangement metathesis as a key step

**DOI:** 10.3762/bjoc.11.188

**Published:** 2015-09-24

**Authors:** Sambasivarao Kotha, Rama Gunta

**Affiliations:** 1 Department of Chemistry, Indian Institute of Technology-Bombay, Powai, Mumbai-400 076, India

**Keywords:** allylation, propellane derivatives, quinones, ring-rearrangement metathesis

## Abstract

Various intricate propellane derivatives and oxa-bowls have been synthesized via a ring-rearrangement metathesis (RRM) as a key step starting from readily accessible starting materials such as *p*-benzoquinone, 1,4-naphthoquinone and 1,4-anthraquinone.

## Introduction

The synthesis of complex target structures requires bond-disconnection analysis of the target molecule, eventually to arrive at simple starting materials by working in an opposite direction to a chemical synthesis. The ‘retrosynthetic analysis’ was first introduced by E. J. Corey and defined as “*it is a problem solving technique for transforming the structure of a synthetic target molecule to a sequence of progressively simpler structures along a pathway which ultimately leads to a simple or commercially available starting materials for a chemical synthesis*” [1] . Generally, this type of retrosynthetic analysis has been used to design [2–6] the target molecule. However, a “transformation-based” retrosynthetic approach is rarely used. In the transformation-based strategy the target and precursor compounds are related by a rearrangement as the key transformation. The advantages of the rearrangement-based strategy are: the target molecule can be assembled from less obvious and more accessible precursors. Several C–C bonds are formed in a simple manner by taking advantage of the key rearrangement and the overall synthetic economy of the process can be enhanced. One can design unprecedented synthetic routes to complex targets [7] through the rearrangement-based approach. In this regard, the ring-rearrangement metathesis (RRM) [8–12] is useful and moreover, the stereochemical information can be transferred from the starting material to the final product during the RRM. In continuation of our interest to design novel molecules via metathesis [13–20] we conceived a new and simple route to propellane derivatives and oxa-bowls [21–26]. This strategy starts from simple starting materials and involves a Diels–Alder (DA) reaction [27–28] and RRM as the key steps.

## Results and Discussion

### Strategy

**Figure 1:**
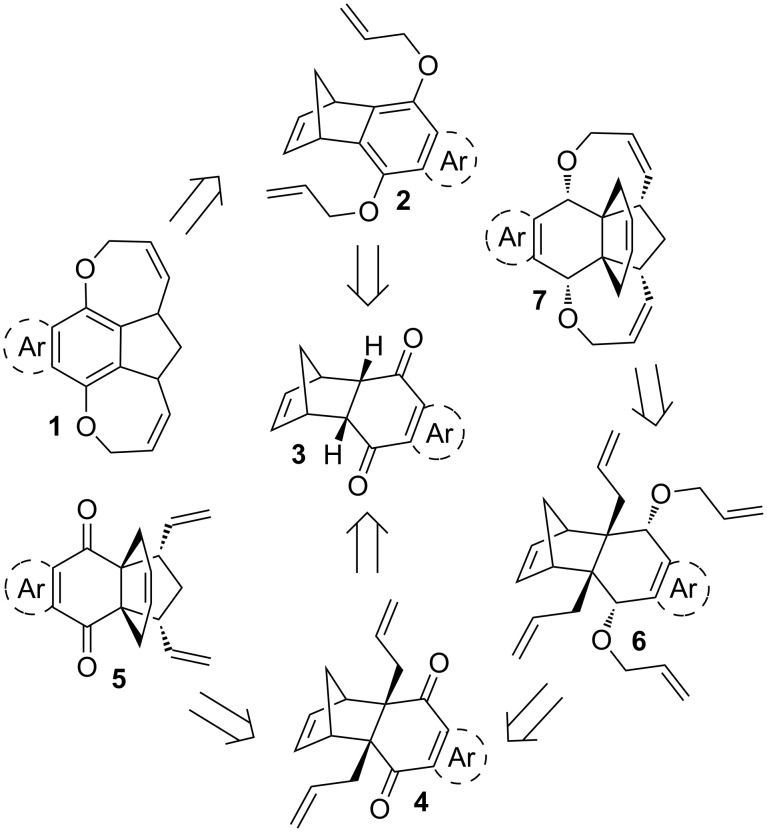
RRM route to propellane derivatives and oxa-bowls.

The retrosynthetic strategy to diverse propellane derivatives and oxa-bowls is shown in Figure 1. Oxa-bowl **1** can be synthesized from the tetracyclic compound **2** using RRM, which could be obtained from the known DA adduct **3** by O-allylation. On the other hand, the propellane derivative **7** may be synthesized from the tetraallyl compound **6** by a RRM sequence. Further, the tetraallyl compound **6** can be assembled from the C-allyl derivative **4** via reduction followed by O-allylation. The C-allyl derivative **4** may be obtained from the known DA adduct **3** by a C-allylation sequence which in turn could be prepared by the DA reaction of the corresponding 1,4-quinones (*p*-benzoquinone, 1,4-naphthoquinone or 1,4-anthraquinone) with a freshly cracked cyclopentadiene.

**Scheme 1:**
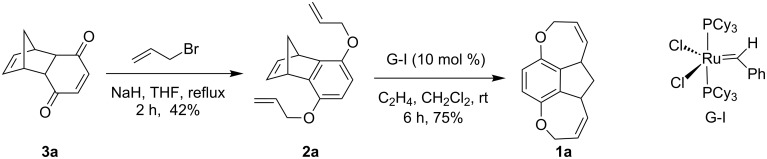
Synthesis of the oxa-bowl **1a** via RRM.

To realize the synthetic strategy (Figure 1) to various propellane derivatives [29–31] and oxa-bowls, we commenced with the preparation of a known DA adduct **3a** [32]. Subsequent allylation of **3a** with allyl bromide in the presence of NaH delivered the aromatized compound **2a** in 42% yield. Then, the tricyclic compound **2a** was subjected to RRM with Grubbs 1^st^ generation (G-I) catalyst in the presence of ethylene to furnish the tetracyclic compound **1a** in 75% yield (Scheme 1). The structures of compounds **2a** and **1a** have been confirmed on the basis of ^1^H, ^13^C NMR and DEPT-135 spectral data and further supported by HRMS data.

**Scheme 2:**
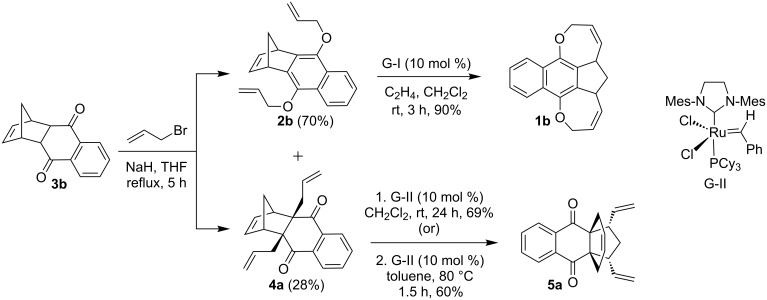
Synthesis of RRM products **1b** and **5a** starting from DA adduct **3b**.

To expand the strategy, another DA adduct **3b** was prepared from the commercially available 1,4-naphthoquinone and freshly cracked cyclopentadiene by following the literature procedure [33]. Allylation of adduct **3b** under similar reaction conditions as described above gave O-allylated compound **2b** and C-allylated compound **4a** in 70% and 28% yields, respectively. Then, treatment of the O-allyl compound **2b** with G-I catalyst in the presence of ethylene at room temperature (rt) produced the RRM product, a pentacyclic oxa-bowl **1b** in 90% yield. When the C-allyl compound **4a** was treated with G-II catalyst in CH_2_Cl_2_ at rt or in refluxing toluene, the propellane derivative **5a** was obtained in 69% yield (Scheme 2). The structures of the new compounds **2b**, **4a**, **1b** and **5a** have been established on the basis of ^1^H and ^13^C NMR spectral data and further supported by HRMS data.

Next, another DA adduct **3c** was prepared from readily available starting materials. In this regard, 1,4-anthraquinone was prepared from quinizarin (1,4-dihydroxyanthraquinone) by using the literature procedure [34] and the known DA adduct **3c** was obtained by a cycloaddition reaction [35] of 1,4-anthraquinone and cyclopentadiene.

**Scheme 3:**
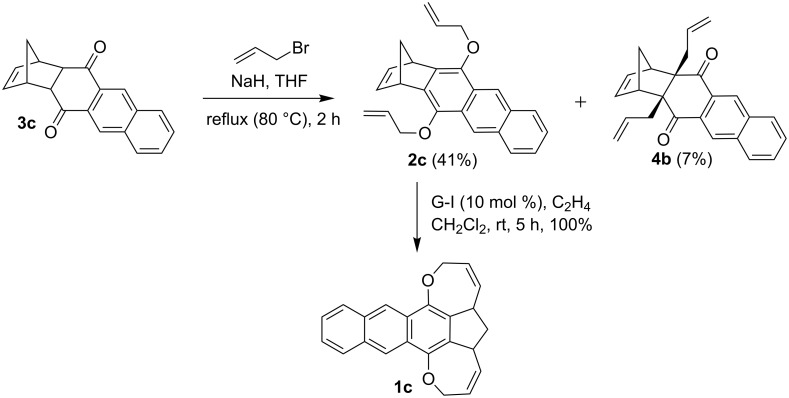
Synthesis of the hexacyclic compound **1c** using RRM.

Again, allylation of the DA adduct **3c** with allyl bromide in the presence of NaH afforded the O-allylated compound **2c** in 41% and the C-allylated compound **4b** in 7% yield. Compound **2c** was further subjected to RRM with G-I catalyst in the presence of ethylene to deliver the hexacyclic oxa-bowl **1c** in quantitative yield (Scheme 3).

Having the C-allylated DA adducts **4a**,**b** in hand, compound **4a** was reduced with diisobutylaluminium hydride (DIBAL-H) at −74 °C to furnish diol **8a** in 81% yield along with a minor amount of compound **9** (8%). The formation of compound **9** may be explained on the basis of a retro-DA reaction [36] followed by reduction and elimination. In the same way, reduction of C-allyl compound **4b** under similar reaction conditions gave diol **8b** in 88% yield.

**Scheme 4:**
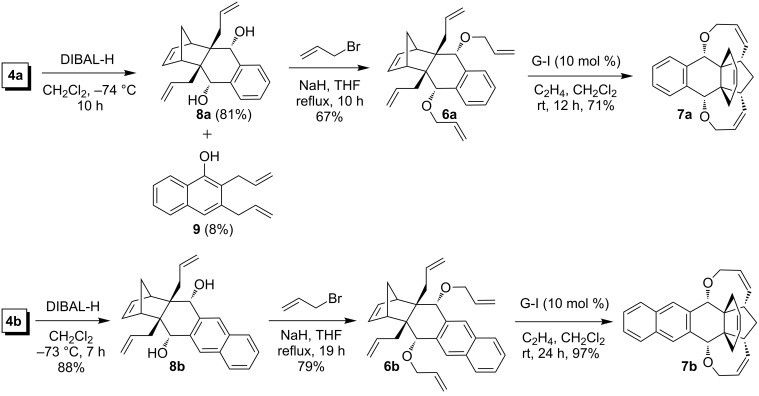
Synthesis of the propellane/oxa-bowl hybrids **7a**,**b** via RRM.

In the next step, diols **8a,b** were O-allylated with allyl bromide in the presence of NaH to furnish the desired RRM precursors **6a**,**b** in 67% and 79% yields respectively (Scheme 4).

Finally, the tetraallyl derivatives **6a**,**b** were subjected to RRM with G-I catalyst in the presence of ethylene at rt to produce the corresponding propellane/oxa-bowl hybrids **7a**,**b** in 71% and 97% yields, respectively. The new compounds **2c**, **4b**, **1c**, **8a**,**b**, **9**, **6a**,**b** and **7a**,**b** have been fully characterized by using spectroscopic techniques (^1^H, ^13^C NMR and DEPT-135) and HRMS data.

## Conclusion

We have successfully synthesized diverse heterocycles **1a–c** in a simple manner starting from the known DA adducts **3a–c**, including the propellane/oxa-bowl hybrids **7a**,**b** and propellane derivative **5a**. Interestingly, the structurally complex propellane/oxa-bowl hybrids **7a**,**b** were obtained through a four step synthetic sequence starting from simple DA adducts **3b**,**c**, which are otherwise difficult to synthesize following conventional retrosynthetic routes. This methodology can easily be extended for diversity-oriented synthesis [37] by employing different dienes and dienophiles during the DA reaction sequence.

## Supporting Information

File 1 Detailed experimental procedures, characterization data and copies of ^1^H and ^13^C NMR for all new compounds.
