# Supplementary material for: Design and synthesis of propellane derivatives and oxa-bowls via ring-rearrangement metathesis as a key step
Source: Beilstein J Org Chem. 2015 Sep 24;11:1727–31. doi: 10.3762/bjoc.11.188 (PMC4660881; doi:10.3762/bjoc.11.188)

Supporting Information

for

Design and synthesis of propellane derivatives and oxa-bowls via ring-rearrangement metathesis as a key step

Sambasivarao Kotha[§]* and Rama Gunta

Address: Department of Chemistry, Indian Institute of Technology-Bombay, Powai, Mumbai, India

[§]Fax: 022-25767152

Email: Sambasivarao Kotha - srk@chem.iitb.ac.in

*Corresponding author

Detailed experimental procedures, characterization data and copies of ^{1}H and ^{13}C NMR for all new compounds

Contents

Experimental procedures and characterization data S2

Copies of ^{1}H and ^{13}C NMR spectra of the all new compounds S11

Experimental procedures and characterization data

General methods

All reactions were monitored by thin-layer chromatography (TLC) using an appropriate mixture of EtOAc and petroleum ether for development. Reactions involving oxygen-sensitive reagents or catalysts were performed in degassed solvents. Transfer of moisture-sensitive materials was carried out using standard syringe–septum techniques and the reactions were maintained under nitrogen or argon atmosphere until work-up. Dry dichloromethane (CH_2Cl_2) was obtained by distillation from P_2O_5 and anhydrous tetrahydrofuran (THF) was obtained by distillation from sodium benzophenone directly prior to use. All commercial grade reagents were used without further purification.

Techniques

Melting points were recorded on a Veego melting point apparatus and are uncorrected. Nuclear Magnetic Resonance (NMR) spectra were generally recorded on a Bruker (Avance^{TM} 400 or Avance^{TM} III 500) spectrometer operating at 400 or 500 MHz for ^{1}H and 100.6 or 125.7 MHz for ^{13}C nuclei. NMR samples were generally made in deutero chloroform as the solvent and chemical shifts (δ values) are reported in parts per million (ppm) using tetramethylsilane (TMS) as an internal standard. Coupling constants (*J*) are reported in hertz (Hz). The standard abbreviations s, d, t, q, m, dd, dt, dq, td and br refer to singlet, doublet, triplet, quartet, multiplet, doublet of doublet, doublet of triplet, doublet of quartet, triplet of doublet and broadened, respectively. The high-resolution mass spectrometric (HRMS) measurements were carried out using a Bruker (Maxis Impact) or Micromass Q-ToF spectrometer. Infrared (IR) spectra were recorded on a Nicolet Impact-400 FT IR spectrometer.

General procedure for the allylation of Diels–Alder adducts 3a–c and diols 8a,b: To a stirred suspension of sodium hydride (10 equiv) in THF, a solution of the DA adducts **3a**–**c**,

8a or **8b** in 5 mL of THF was added at 0 °C under nitrogen atmosphere and the reaction mixture was stirred at room temperature (rt) for 10 min. Afterwards, allyl bromide (3 equiv) was added and the resulting reaction mixture was refluxed (70–80 °C) for 2–19 h. After completion of the reaction (TLC), the reaction mixture was quenched with saturated NH_4Cl solution (5–15 mL) and extracted with EtOAc. The combined organic layers were washed with brine, dried over anhydrous Na_2SO_4 and concentrated in vacuo. The crude products were purified by silica gel column chromatography using an appropriate mixture of EtOAc and petroleum ether (1–3% EtOAc–petroleum ether) or pure petroleum ether to obtain compounds **2a**–**c** in 41–70%, **4a**,**b** in 7–28% and **6a**,**b** in 67–79% yield.

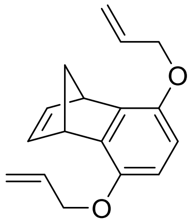

Compound 2a: Obtained from **3a** (100 mg, 0.57 mmol), THF (15 mL); yellow liquid (62 mg, 42%); ^1H NMR (400 MHz, $CDCl_3$): δ (ppm) = 6.85 (t, J = 1.8 Hz, 2H), 6.51 (s, 2H), 6.13–6.04 (m, 2H), 5.42 (dq, J = 17.2, 1.5 Hz, 2H), 5.28 (dd, J = 10.5, 1.4 Hz, 2H) 4.51 (dt, J = 5.2, 1.3 Hz, 4H), 4.22–4.21 (m, 2H), 2.26–2.20 (m, 2H); ^{13}C NMR (100.6 MHz, $CDCl_3$): δ (ppm) = 148.1 (s), 143.1 (d), 141.2 (s), 134.2 (d), 117.2 (t), 111.7 (d), 70.5 (t), 70.0 (t), 47.2 (d); HRMS (ESI, Q-ToF) *m/z*: calculated for $C_{17}H_{18}NaO_2$ $[M+Na]^+$: 277.1199, found: 277.1195; IR (neat): υ_{max} = 3077, 2937, 2871, 1492, 1258, 1011, 915 cm^{-1}.

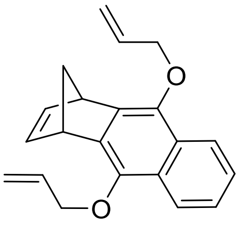

Compound 2b: Obtained from **3b** (2 g, 8.92 mmol), THF (30 mL); yellow thick liquid (1.90 g, 70%); ^1H NMR (400 MHz, $CDCl_3$): δ (ppm) = 8.04 (dd, J = 6.3, 3.3 Hz, 2H), 7.43 (dd, J = 6.3, 3.3 Hz, 2H), 6.76 (t, J = 1.7 Hz, 2H), 6.23–6.13 (m, 2H), 5.45 (dq, J = 17.1, 1.5 Hz, 2H), 5.30 (dq, J = 10.4, 1.2 Hz, 2H), 4.59 (qdt, J = 12.5, 5.6, 1.3 Hz, 4H), 4.28 (t, J = 1.8 Hz, 2H), 2.27 (dt, J = 7.5, 1.5 Hz, 1H), 2.17 (d, J = 7.5 Hz, 1H); ^{13}C NMR (100.6 MHz, $CDCl_3$): δ (ppm) = 144.2, 141.9, 136.7, 134.4, 128.3, 125.6, 122.3, 117.7, 75.5, 65.4, 47.1; HRMS

(ESI, Q-ToF) *m/z*: calculated for $C_{21}H_{20}NaO_2$ $[M+Na]^+$: 327.1356, found: 327.1356; IR (neat): υ_{max} = 3068, 2951, 2878, 1644, 1336, 1082, 769 cm^{-1}.

Compound 4a: Orange thick liquid (758 mg, 28%)

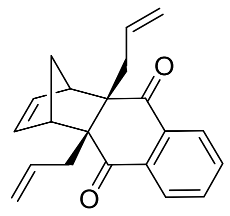

^1H NMR (400 MHz, $CDCl_3$): δ (ppm) = 7.83 (dd, *J* = 5.8, 3.3 Hz, 2H) 7.68 (dd, *J* = 5.7, 3.3 Hz, 2H) 6.42 (t, *J* = 1.9 Hz, 2H), 5.79–5.69 (m, 2H), 5.04–5.01 (m, 2H), 4.94 (dq, *J* = 16.9, 1.4 Hz, 2H), 3.61–3.59 (m, 2H), 2.35–2.29 (m, 2H), 2.16 (dd, *J* = 14.3, 7.2 Hz, 2H), 1.51 (d, *J* = 9.4 Hz, 1H), 1.11 (d, *J* = 9.4 Hz, 1H); ^{13}C NMR (100.6 MHz, $CDCl_3$): δ (ppm) = 200.1, 137.5, 137.1, 134.2, 133.8, 126.6, 118.0, 61.5, 50.5, 47.5, 38.7; HRMS (ESI, Q-ToF) *m/z*: calculated for $C_{21}H_{20}NaO_2$ $[M+Na]^+$: 327.1356, found: 327.1358; IR (neat): υ_{max} = 3073, 2929, 2869, 1682, 1450, 1270, 917 cm^{-1}.

Compound 2c: Obtained from **3c** (330 mg, 1.20 mmol), THF (30 mL); off-white solid (174 mg, 41%), mp: 90–92 °C; ^1H NMR (400 MHz, $CDCl_3$): δ (ppm) = 8.56 (d, *J* = 5.2 Hz, 2H), 8.02–7.99 (m, 2H), 7.46–7.43 (m, 2H), 6.73 (br s, 2H), 6.30–6.20 (m, 2H), 5.52–5.47 (m, 2H), 5.35–5.32 (m, 2H), 4.72–4.64 (m, 4H), 4.31 (br s, 2H), 2.27 (d, *J* = 7.6 Hz, 1H), 2.16 (d, *J* = 7.5 Hz, 1H); ^{13}C NMR (100.6 MHz, $CDCl_3$): δ (ppm) = 143.6 (s), 141.2 (d), 134.5 (d), 134.3 (s), 131.6 (s), 128.5 (d), 127.8 (s), 125.4 (d), 121.0 (d), 117.9 (t), 75.5 (t), 63.2 (t), 46.8 (d); HRMS (ESI, Q-ToF) *m/z*: calculated for $C_{25}H_{23}O_2$ $[M+H]^+$: 355.1693, found: 355.1694; IR (neat): υ_{max} = 3050, 2991, 2863, 1655, 1447, 1319, 1010, 750 cm^{-1}.

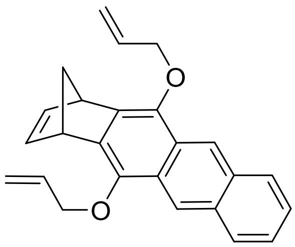

Compound 4b: Yellow solid (32 mg, 7%), mp: 113–115 °C

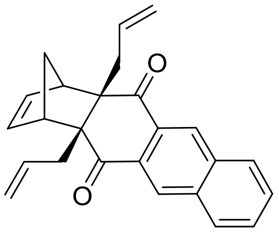

^1H NMR (500 MHz, $CDCl_3$): δ (ppm) = 8.37 (s, 2H), 8.02 (dd, *J* = 6.2, 3.3 Hz, 2H), 7.65 (dd, *J* = 6.3, 3.2 Hz, 2H), 6.45 (t, *J* = 1.7 Hz, 2H), 5.83–5.75 (m, 2H), 5.04–5.02 (m, 2H), 4.96 (dd, *J* = 16.9, 1.5 Hz, 2H), 3.66 (t, *J* = 1.6 Hz, 2H), 2.34 (dd, *J* = 14.4, 7.0 Hz, 2H), 2.17 (dd, *J* = 14.3, 7.2 Hz,

2H), 1.55 (d, J = 9.5 Hz, 1H), 1.19 (d, J = 9.4 Hz, 1H); ^{13}C NMR (100.6 MHz, $CDCl_3$): δ (ppm) = 200.3 (s), 137.2 (d), 135.3 (s), 134.4 (d), 133.7 (s), 129.8 (d), 129.1 (d), 127.9 (d), 117.9 (t), 61.7 (s), 50.6 (d), 47.7 (t), 38.9 (t); HRMS (ESI, Q-ToF) *m/z*: calculated for $C_{25}H_{22}NaO_2$ $[M+Na]^+$: 377.1512, found: 377.1513; IR (neat): υ_{max} = 3061, 2975, 2867, 1682, 1455, 1265, 911 cm^{-1}.

Compound 6a: Obtained from **8a** (255 mg, 0.83 mmol), THF (30 mL); colourless thick liquid (215 mg, 67%); ^{1}H NMR (500 MHz, $CDCl_3$): δ (ppm) = 7.36–7.34 (m, 2H), 7.24–7.23 (m, 2H), 6.26 (s, 2H), 6.07–5.99 (m, 2H), 5.96–5.88 (m, 2H), 5.40 (dd, J = 17.2, 1.3 Hz, 2H), 5.23 (dd, J = 10.5, 0.8 Hz, 2H), 4.78–4.71 (m, 4H), 4.53 (s, 2H), 4.35 (dd, J = 13.0, 4.7 Hz, 2H), 4.08 (dd, J = 13.0, 5.7 Hz, 2H), 3.04 (s, 2H), 2.25 (d, J = 9.6 Hz, 1H), 2.07 (dd, J = 14.9, 8.4 Hz, 2H), 1.75 (dd, J = 14.9, 5.2 Hz, 2H), 1.54 (d, J = 9.6 Hz, 1H); ^{13}C NMR (100.6 MHz, $CDCl_3$): δ (ppm) = 139.7 (d), 138.1 (s), 136.9 (d), 135.2 (d), 126.3 (d), 123.5 (d), 116.8 (t), 114.6 (t), 82.5 (d), 72.8 (t), 57.0 (s), 50.5 (d), 43.5 (t), 37.4 (t); HRMS (ESI, Q-ToF) *m/z*: calculated for $C_{27}H_{32}NaO_2$ $[M+Na]^+$: 411.2295, found: 411.2296; IR (neat): υ_{max} = 3072, 2964, 2856, 1635, 1456, 1117, 1067, 915 cm^{-1}.

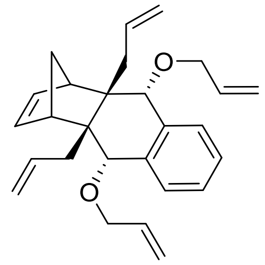

Compound 6b: Obtained from **8b** (79 mg, 0.22 mmol), THF (10 mL); colourless liquid (76 mg, 79%); ^{1}H NMR (400 MHz, $CDCl_3$): δ (ppm) = 7.87 (dd, J = 6.2, 3.2 Hz, 2H), 7.79 (s, 2H), 7.47 (dd, J = 6.2, 3.2 Hz, 2H), 6.30 (t, J = 1.8 Hz, 2H), 6.16–5.98 (m, 4H), 5.46 (dq, J = 17.2, 1.7 Hz, 2H), 5.30 (dq, J = 10.5, 1.4 Hz, 2H), 4.80–4.72 (m, 6H), 4.48 (ddt, J = 13.0, 4.8, 1.6 Hz, 2H), 4.17 (ddt, J = 13.1, 5.8, 1.4 Hz, 2H), 3.14 (t, J = 1.6 Hz, 2H), 2.30 (d, J = 9.7 Hz, 1H), 2.30 (dd, J = 15.0, 8.5 Hz, 2H), 1.77 (dd, J = 14.9, 5.2 Hz, 2H), 1.61 (d, J = 9.7 Hz, 1H); ^{13}C NMR (100.6 MHz, $CDCl_3$): δ (ppm) = 139.8 (d), 136.8 (d), 136.5 (s), 135.2 (d), 132.5 (s), 128.0 (d), 125.6 (d), 122.3 (d), 117.0 (t), 114.5 (t), 82.5 (d), 73.0 (t), 56.4 (s),

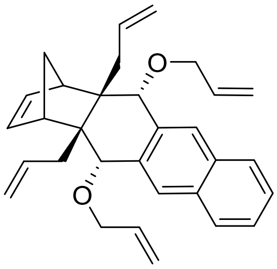

50.2 (d), 43.4 (t), 37.0 (t); HRMS (ESI, Q-ToF) *m/z*: calculated for $C_{31}H_{34}NaO_2$ $[M+Na]^+$: 461.2451, found: 461.2454; IR (neat): υ_{max} = 3070, 2971, 2855, 1633, 1445, 1418, 1112, 1068, 901 cm^{-1}.

General procedure for the RRM of the *O*-allyl compounds 2a–c and 6a,b: The solution of **2a**–**c**, **6a** or **6b** in dry CH_2Cl_2 was degassed with nitrogen for 5 min and purged with ethylene gas for another 10 min. Afterwards, Grubbs 1st generation (G-I) catalyst (10 mol %) was added in the presence of ethylene gas and the resulting reaction mixture was stirred at rt for 3–24 h. After completion of the reaction (TLC), the solvent was removed and the crude product was purified by silica gel column chromatography using an appropriate mixture of EtOAc and petroleum ether (1–4% EtOAc–petroleum ether) to obtain the desired oxa-bowls **1a**–**c** or the propellane derivatives **7a**,**b** in 71–100% yield.

Compound 1a: Obtained from **2a** (42 mg, 0.16 mmol), CH_2Cl_2 (35 mL); pale yellow solid (28 mg, 75%), mp: decomposed >95 ^oC; ^1H NMR (400 MHz, $CDCl_3$): δ (ppm) = 6.70 (s, 2H), 5.81 (d, J = 11.1 Hz, 2H), 5.75–5.69 (m, 2H), 4.77 (dd, J = 15.3, 5.6 Hz, 2H), 4.41 (d, J = 15.2 Hz, 2H), 4.23–4.19 (m, 2H) 2.67–2.60 (m, 1H), 1.66, 1.60 (ABq, J_{AB} = 11.8 Hz, 1H); ^{13}C NMR (100.6 MHz, $CDCl_3$): δ (ppm) = 152.3 (s), 135.2 (s), 133.6 (d), 126.6 (d), 119.4 (d), 68.8 (t), 42.7 (d), 41.2 (t); HRMS (ESI, Q-ToF) *m/z*: calculated for $C_{15}H_{15}O_2$ $[M+H]^+$: 227.1067, found: 227.1072; IR (neat): υ_{max} = 3015, 2980, 2866, 1485, 1228, 1052, 752 cm^{-1}.

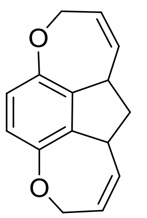

Compound 1b: Obtained from **2b** (100 mg, 0.33 mmol), CH_2Cl_2 (70 mL); yellow sticky solid (82 mg, 90%); ^1H NMR (400 MHz, $CDCl_3$): δ (ppm) = 8.13 (dd, J = 6.3, 3.4 Hz, 2H), 7.45 (dd, J = 6.4, 3.3 Hz, 2H), 5.91–5.88 (m, 2H), 5.85–5.79 (m, 2H), 4.98 (dd, J = 15.2, 5.0 Hz, 2H), 4.57–4.53 (m, 2H), 4.38–4.32 (m, 2H), 2.72–2.66 (m, 1H), 1.72, 1.66 (ABq, J_{AB} = 12.0 Hz, 1H); ^{13}C NMR (100.6 MHz, $CDCl_3$): δ (ppm) = 146.9, 133.3, 130.2, 127.6, 126.8, 125.5, 121.7, 68.8, 42.5, 41.8;

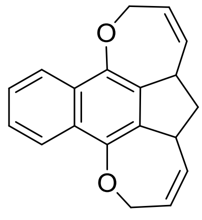

HRMS (ESI, Q-ToF) *m/z*: calculated for $C_{19}H_{17}O_2$ $[M+H]^+$: 277.1223, found: 277.1221; IR (neat): υ_{max} = 3014, 2929, 1640, 1605, 1456, 1349, 1097 cm^{-1}.

Compound 1c: Obtained from **2c** (150 mg, 0.42 mmol), CH_2Cl_2 (40 mL); yellow sticky solid (138 mg, 100%), mp: 152–154 °C (decomposed); ^1H NMR (400 MHz, $CDCl_3$): δ (ppm) = 8.69 (s, 2H), 8.01 (dd, J = 6.4, 3.3 Hz, 2H), 7.44 (dd, J = 6.5, 3.2 Hz, 2H), 5.97–5.94 (m, 2H), 5.92–5.86 (m, 2H), 5.11–5.05 (m, 2H), 4.66–4.60 (m, 2H), 4.42–4.36 (m, 2H), 2.73–2.66 (m, 1H), 1.76, 1.70 (ABq, J_{AB} = 12.1 Hz, 1H); ^{13}C NMR (100.6 MHz, $CDCl_3$): δ (ppm) = 146.5 (s), 133.8 (d), 131.5 (s), 128.6 (d), 128.2 (s), 127.0 (s), 126.9 (d), 125.4 (d), 120.4 (d), 68.6 (t), 42.4 (d), 41.7 (t); HRMS (ESI, Q-ToF) *m/z*: calculated for $C_{23}H_{18}KO_2$ $[M+K]^+$: 365.0938, found: 365.0936; IR (neat): υ_{max} = 3013, 2941, 2862, 1643, 1451, 1344, 1304, 1087, 986 cm^{-1}.

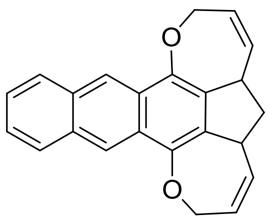

Compound 7a: Obtained from **6a** (27 mg, 0.07 mmol), CH_2Cl_2 (30 mL); white solid (16.50 mg, 71%), mp: 162–163 °C; ^1H NMR (400 MHz, $CDCl_3$): δ (ppm) = 7.33–7.30 (m, 2H), 7.20 (dd, J = 5.5, 3.4 Hz, 2H), 6.12–6.08 (m, 2H), 6.05–5.99 (m, 2H), 5.28 (t, J = 3.4 Hz, 2H), 4.60–4.55 (m, 4H), 4.27 (ddd, J = 14.3, 4.2, 2.0 Hz, 2H), 3.20–3.16 (m, 2H), 2.07 (m, 1H), 1.95–1.83 (m, 5H); ^{13}C NMR (125.7 MHz, $CDCl_3$): δ (ppm) = 138.0 (s), 137.5 (d), 128.4 (d), 127.8 (d), 126.3 (d), 121.8 (d), 88.9 (d), 68.7 (t), 54.8 (d), 52.3 (s), 34.5 (t), 27.2 (t); HRMS (ESI, Q-ToF) *m/z*: calculated for $C_{23}H_{24}KO_2$ $[M+K]^+$: 371.1408, found: 371.1409; IR (neat): υ_{max} = 3022, 2951, 2829, 1459, 1448, 1370, 1084, 892, 766 cm^{-1}.

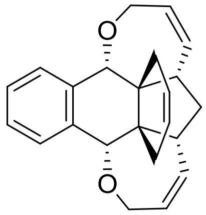

Compound 7b: Obtained from **6b** (71 mg, 0.16 mmol), CH_2Cl_2 (40 mL); white solid (60 mg, 97%), mp: 193–197 °C; ^1H NMR (400 MHz, $CDCl_3$): δ (ppm) = 7.81 (dd, J = 6.1, 3.3 Hz, 2H), 7.73 (s, 2H), 7.41 (dd, J = 6.2, 3.2 Hz, 2H), 6.15–6.11 (m, 2H), 6.08–6.02 (m, 2H), 5.17 (t, J = 3.5 Hz,

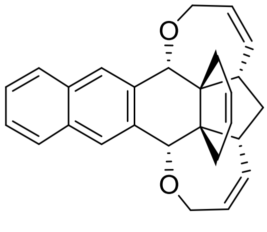

2H), 4.77 (s, 2H), 4.65 (ddd, J = 14.2, 6.8, 0.6 Hz, 2H), 4.37–4.32 (m, 2H), 3.26–3.23 (m, 2H), 2.13–1.93 (m, 4H), 1.83 (d, J = 2.8 Hz, 1H), 1.79 (d, J = 2.8 Hz, 1H); ^{13}C NMR (100.6 MHz, $CDCl_3$): δ (ppm) = 137.5 (d), 136.7 (s), 132.4 (s), 128.4 (d), 128.0 (d), 127.7 (d), 125.5 (d), 120.6 (d), 88.6 (d), 68.7 (t), 54.8 (d), 52.1 (s), 34.2 (t), 27.0 (t); HRMS (ESI, Q-ToF) *m/z*: calculated for $C_{27}H_{26}NaO_2$ $[M+Na]^+$: 405.1825, found: 405.1826; IR (neat): υ_{max} = 3023, 2952, 2833, 1456, 1444, 1366, 1091, 890, 729 cm^{-1}.

Synthesis of the propellane derivative 5a: A solution of **4a** (55 mg, 0.18 mmol) in dry CH_2Cl_2 (50 mL) was degassed with nitrogen for 5 min. Afterwards, Grubbs 2nd generation (G-II) catalyst (15.35 mg, 10 mol %) was added and the resulting mixture was stirred at rt for 24 h. After completion of the reaction (TLC), the solvent was removed and the crude product was purified by silica gel column chromatography (1% EtOAc–petroleum ether) to obtain propellane derivative **5a** as a yellow liquid in 69% yield.

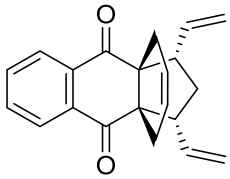

^{1}H NMR (500 MHz, $CDCl_3$): δ (ppm) = 8.09 (dd, J = 5.8, 3.3 Hz, 2H), 7.74 (dd, J = 5.8, 3.3 Hz, 2H), 5.77–5.70 (m, 2H), 5.66 (br s, 2H), 5.05–4.99 (m, 4H), 3.34, 3.30 (ABq, J_{AB} = 8.2 Hz, 2H), 2.62–2.58 (m, 2H), 2.35–2.29 (m, 1H), 2.00 (d, J = 16.3 Hz, 2H), 1.78–1.72 (m, 1H); ^{13}C NMR (125.7 MHz, $CDCl_3$): δ (ppm) = 199.0, 138.2, 134.6, 132.3, 127.6, 125.4, 117.4, 61.0, 49.4, 34.3, 26.1; HRMS (ESI, Q-ToF) *m/z*: calculated for $C_{21}H_{20}NaO_2$ $[M+Na]^+$: 327.1356, found: 327.1359; IR (neat): υ_{max} = 3073, 2929, 1683, 1639, 1595, 1426, 1262, 918 cm^{-1}.

General procedure for the reduction of the ketones 4a,b: To a solution of the ketone **4a** or **4b** in dry CH_2Cl_2 (10–20 mL) was added diisobutylaluminium hydride (DIBAL-H, 1 M solution in toluene) at −73 to −74 °C and stirring was continued at the same temperature for 7–10 h. After completion of the reaction (TLC), the reaction was quenched by the addition of saturated sodium potassium tartrate solution (10–15 mL). Then, the reaction mixture was extracted with EtOAc (3 × 20 mL), the combined organic layers were washed with brine (2 ×

20 mL) and dried over anhydrous Na_2SO_4. The solvent was evaporated in vacuo and the crude product was purified by silica gel column chromatography to obtain the desired diols **8a** along with a minor amount of **9** (19 mg, 8%) or **8b** in 81–88% yield.

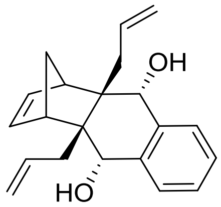

Compound 8a: Obtained from **4a** (300 mg, 0.98 mmol), DIBAL-H (5.91 mL, 6 equiv); white solid (246 mg, 81%), mp: 140–142 ^{o}C; ^{1}H NMR (400 MHz, $CDCl_3$): δ (ppm) = 7.32–7.29 (m, 2H), 7.26–7.24 (m, 2H), 6.37 (s, 2H), 6.16–6.07 (m, 2H), 5.25–5.16 (m, 4H), 4.60 (s, 2H), 2.71 (dd, *J* = 12.8, 7.7 Hz, 2H), 2.51 (s, 2H), 1.95 (dd, *J* = 12.9, 6.9 Hz, 2H), 0.65 (d, *J* = 9.5 Hz, 1H), 0.26 (d, *J* = 9.6 Hz, 1H); ^{13}C NMR (125.7 MHz, $CDCl_3$): δ (ppm) = 141.0 (s), 138.7 (d), 136.4 (d), 129.0 (d), 128.1 (d), 117.5 (t), 75.4 (d), 51.5 (s), 50.7 (d), 44.0 (t), 37.9 (t); HRMS (ESI, Q-ToF) *m/z*: calculated for $C_{21}H_{24}NaO_2$ $[M+Na]^+$: 331.1669, found: 331.1664; IR (neat): υ_{max} = 3177, 2960, 1636, 1456, 1252, 1006, 917 cm^{-1}.

Compound 9: Off-white solid (19 mg, 8%), mp: 94–96 ^{o}C

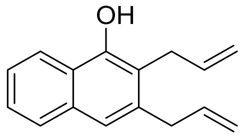

^{1}H NMR (400 MHz, $CDCl_3$): δ (ppm) = 8.13–8.10 (m, 1H), 7.75–7.71 (m, 1H), 7.45–7.41 (m, 2H), 7.29 (s, 1H), 6.10–5.99 (m, 2H), 5.21–5.09 (m, 3H), 5.04–4.98 (m, 1H), 3.59 (dt, *J* = 5.7, 1.8 Hz, 2H), 3.54–3.53 (m, 2H); ^{13}C NMR (100.6 MHz, $CDCl_3$): δ (ppm) = 150.2 (s), 137.3 (d), 137.1 (s), 135.9 (d), 133.4 (s), 127.3 (d), 126.1 (d), 124.9 (d), 123.9 (s), 121.4 (d), 120.9 (d), 117.9 (s), 116.6 (t), 116.3 (t), 38.4 (t), 31.0 (t); HRMS (ESI, Q-ToF) *m/z*: calculated for $C_{16}H_{17}O$ $[M+H]^+$: 225.1274, found: 225.1272; IR (neat): υ_{max} = 3495, 3076, 2924, 1660, 1596, 1293, 915, 752 cm^{-1}.

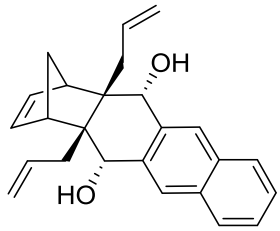

Compound 8b: Obtained from **4b** (100 mg, 0.28 mmol), DIBAL-H (2.52 mL, 9 equiv); pale yellow liquid (89 mg, 88%); ^{1}H NMR (400 MHz, $CDCl_3$): δ (ppm) = 7.78 (dd, *J* = 6.0, 3.2 Hz, 2H), 7.67 (s, 2H), 7.47 (dd, *J* = 6.0, 3.2 Hz, 2H), 6.37 (s, 2H), 6.20–6.09 (m, 2H), 5.27–5.19 (m, 4H),

4.76 (s, 2H), 3.32 (br s, 2H), 2.73 (dd, J = 13.2, 7.6 Hz, 2H), 2.54 (br s, 2H), 1.96 (dd, J = 13.2, 7.2 Hz, 2H), 0.60 (d, J = 9.6 Hz, 1H), 0.26 (d, J = 10.0 Hz, 1H); ^{13}C NMR (100.6 MHz, $CDCl_3$): δ (ppm) = 138.7 (d), 138.6 (d), 136.3 (s), 133.6 (s), 128.0 (d), 126.9 (d), 126.5 (d), 117.6 (t), 75.6 (d), 51.7 (s), 50.7 (d), 44.1 (t), 37.8 (t); HRMS (ESI, Q-ToF) *m/z*: calculated for $C_{25}H_{26}NaO_2$ $[M+Na]^+$: 381.1825, found: 381.1826; IR (neat): υ_{max} = 3328, 2963, 1637, 1451, 1266, 1007, 912 cm^{-1}.

Copies of ^{1}H and ^{13}C NMR spectra of the all new compounds including DEPT-135

Compound 2a: ^{1}H NMR (400 MHz, $CDCl_3$)

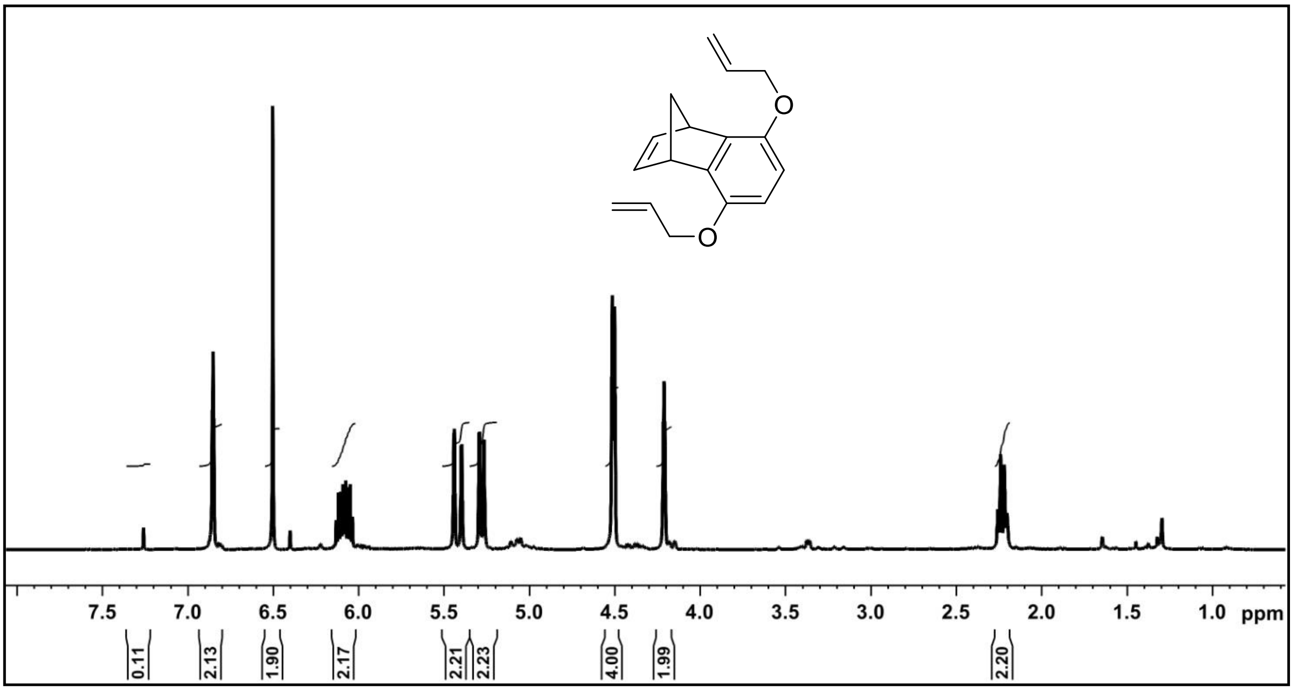

Compound 2a: ^{13}C NMR (100.6 MHz, $CDCl_3$)

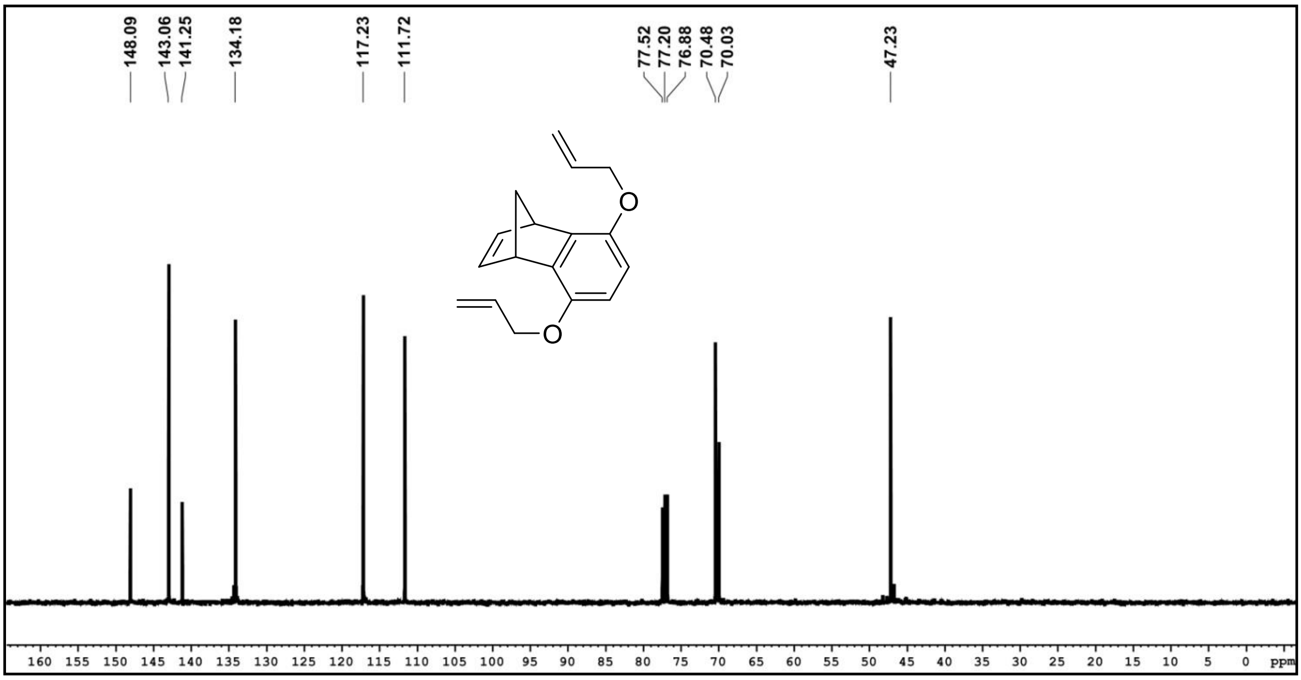

Compound 2a: DEPT-135 NMR (100.6 MHz, $CDCl_3$)

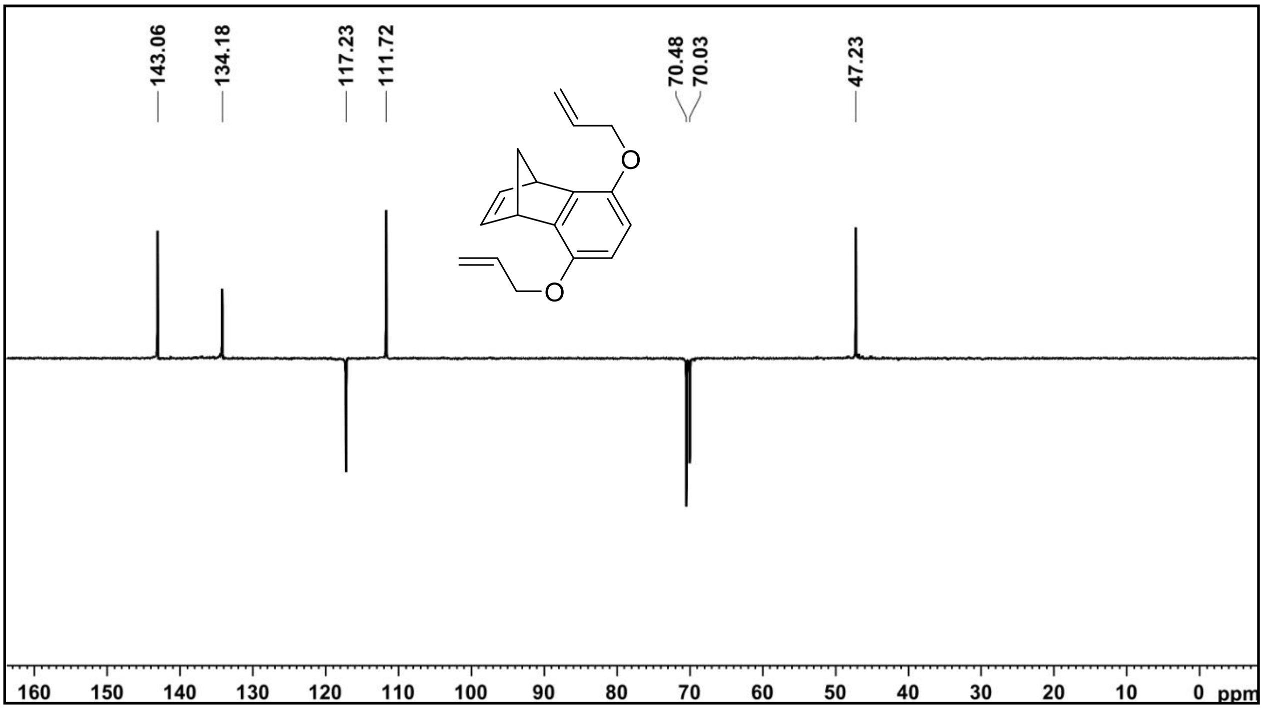

Compound 2b: ^{1}H NMR (400 MHz, $CDCl_3$)

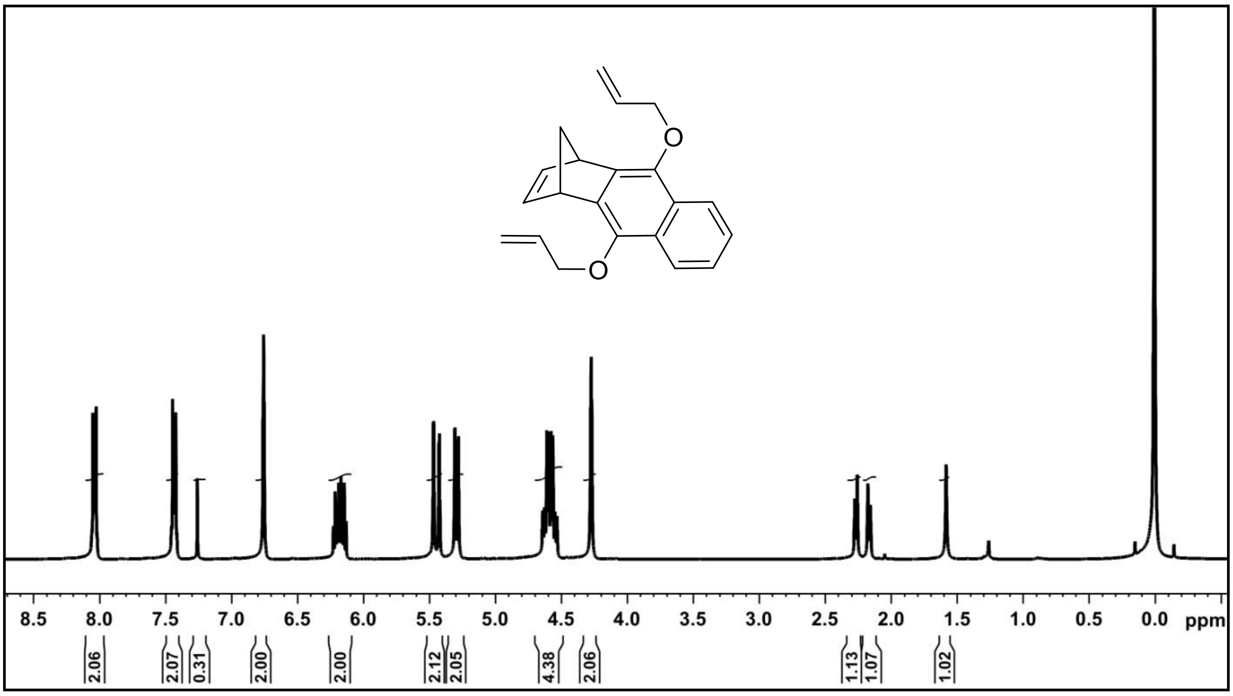

Compound 2b: ^{13}C NMR (100.6 MHz, $CDCl_3$)

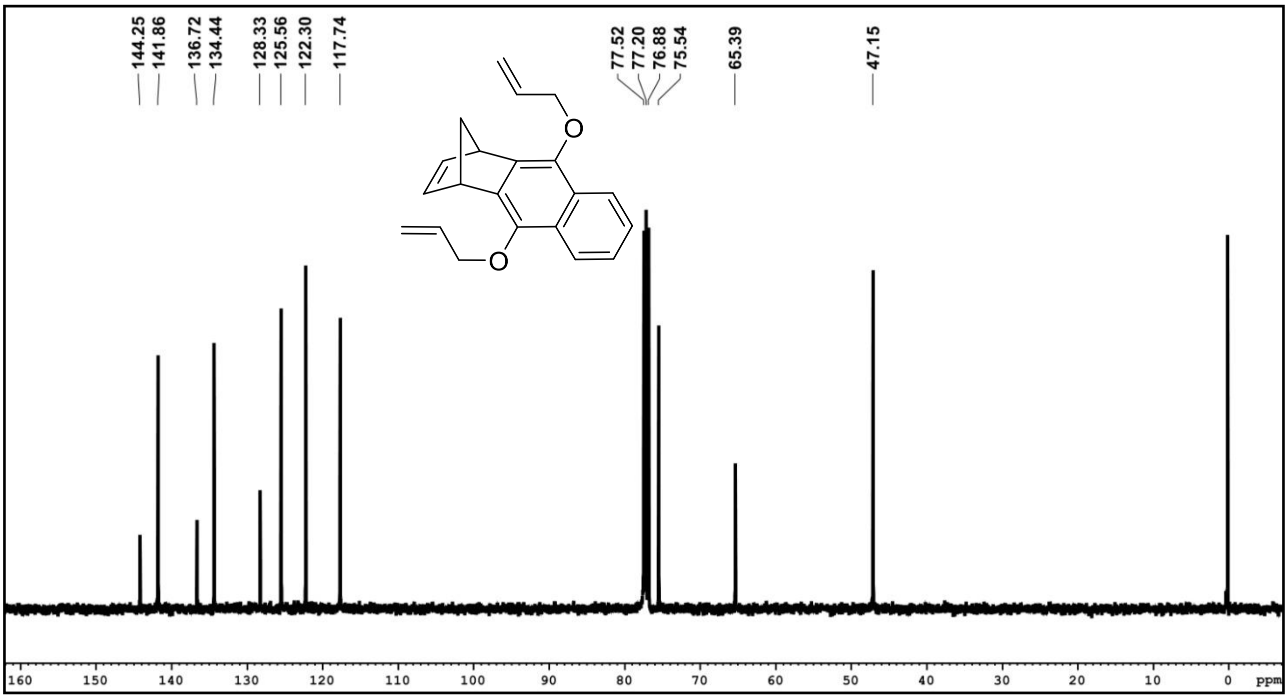

Compound 4a: ^{1}H NMR (400 MHz, $CDCl_3$)

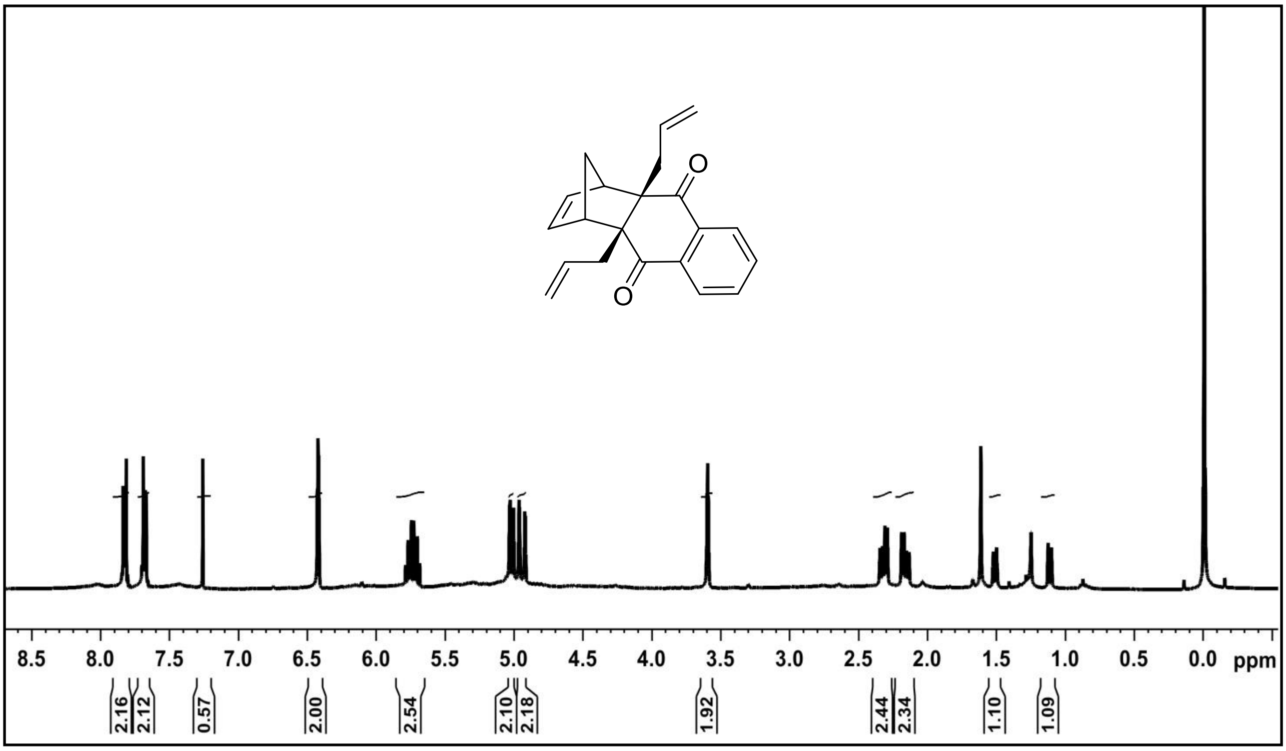

Compound 4a: ^{13}C NMR (100.6 MHz, $CDCl_3$)

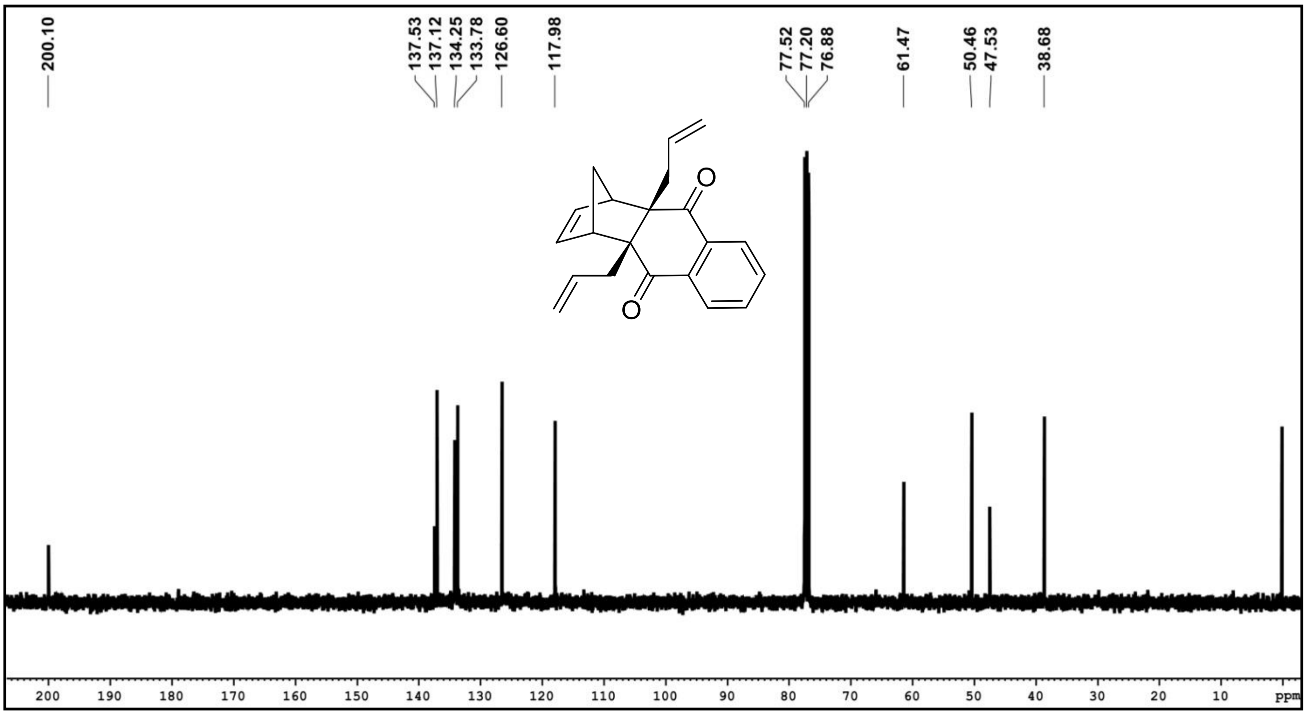

Compound 2c: ^{1}H NMR (400 MHz, $CDCl_3$)

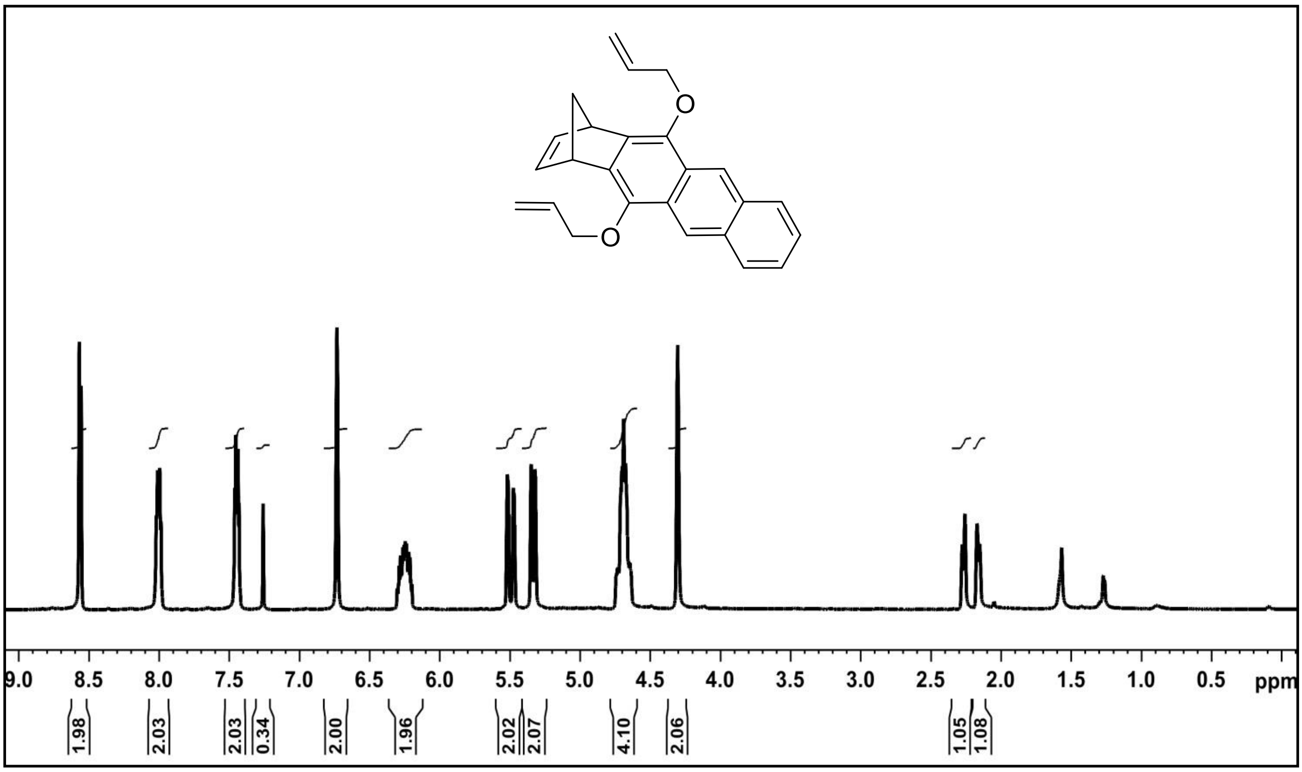

Compound 2c: ^{13}C NMR (100.6 MHz, $CDCl_3$)

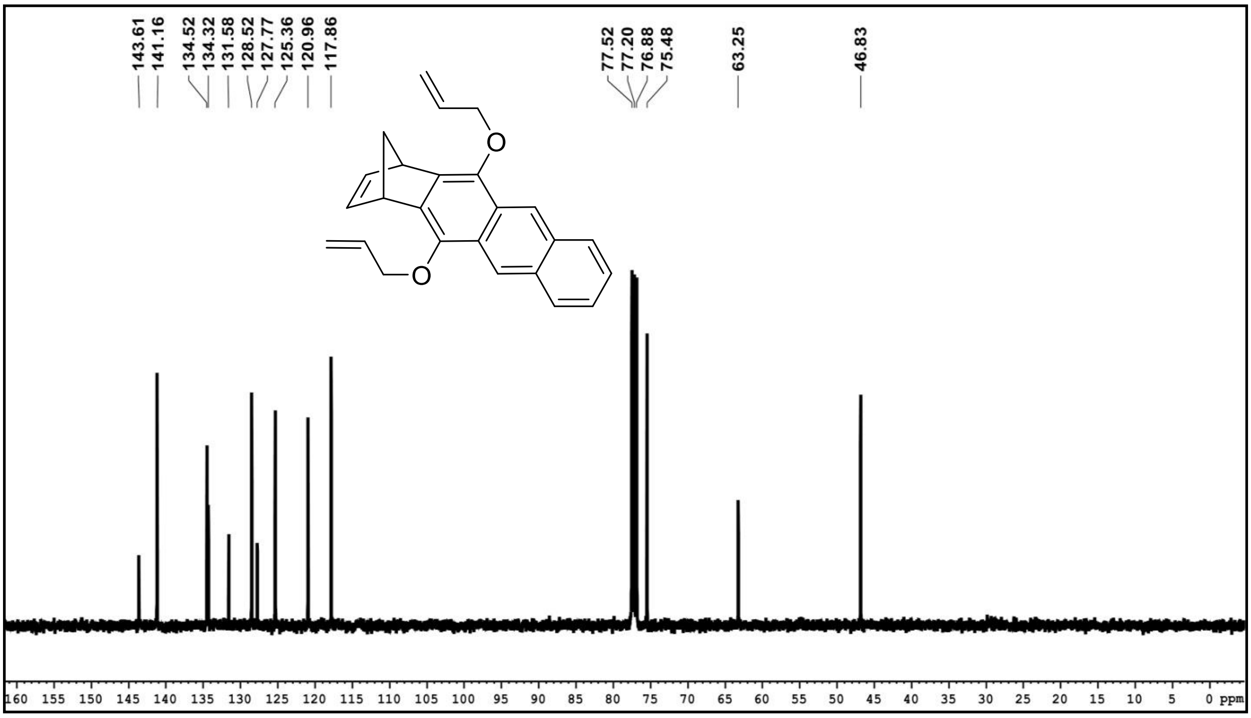

Compound 2c: DEPT-135 NMR (100.6 MHz, $CDCl_3$)

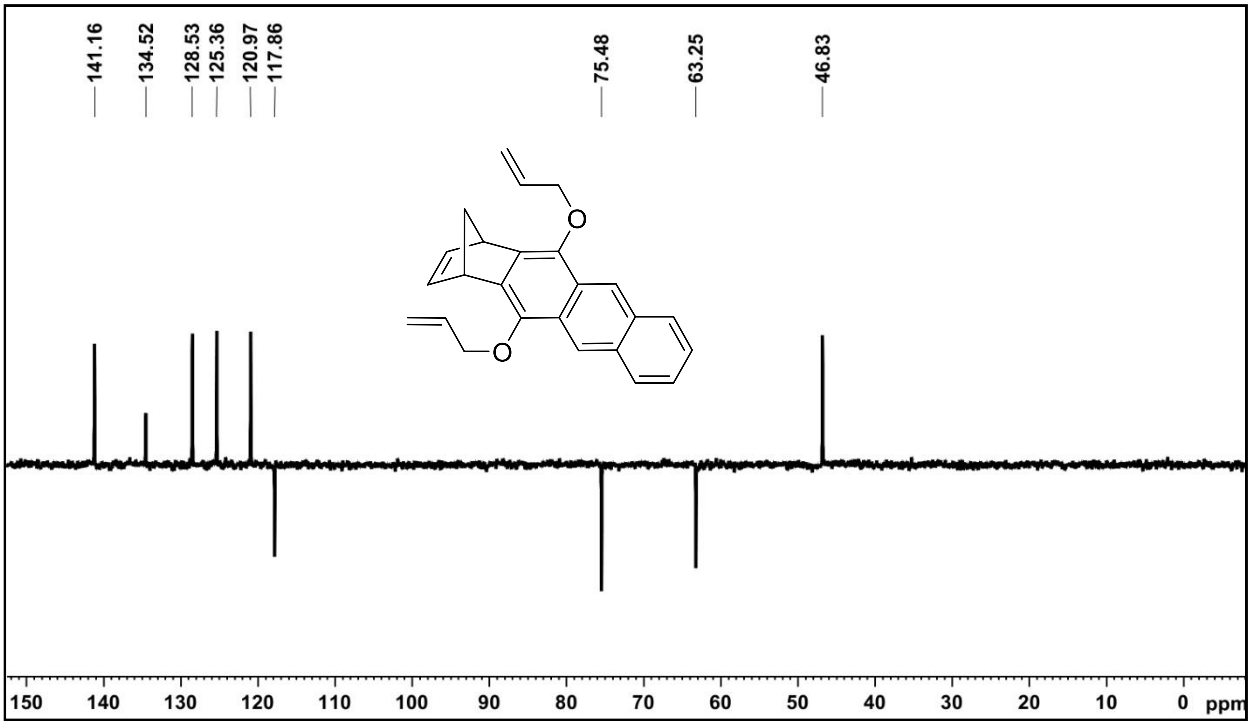

Compound 4b: ^{1}H NMR (500 MHz, $CDCl_3$)

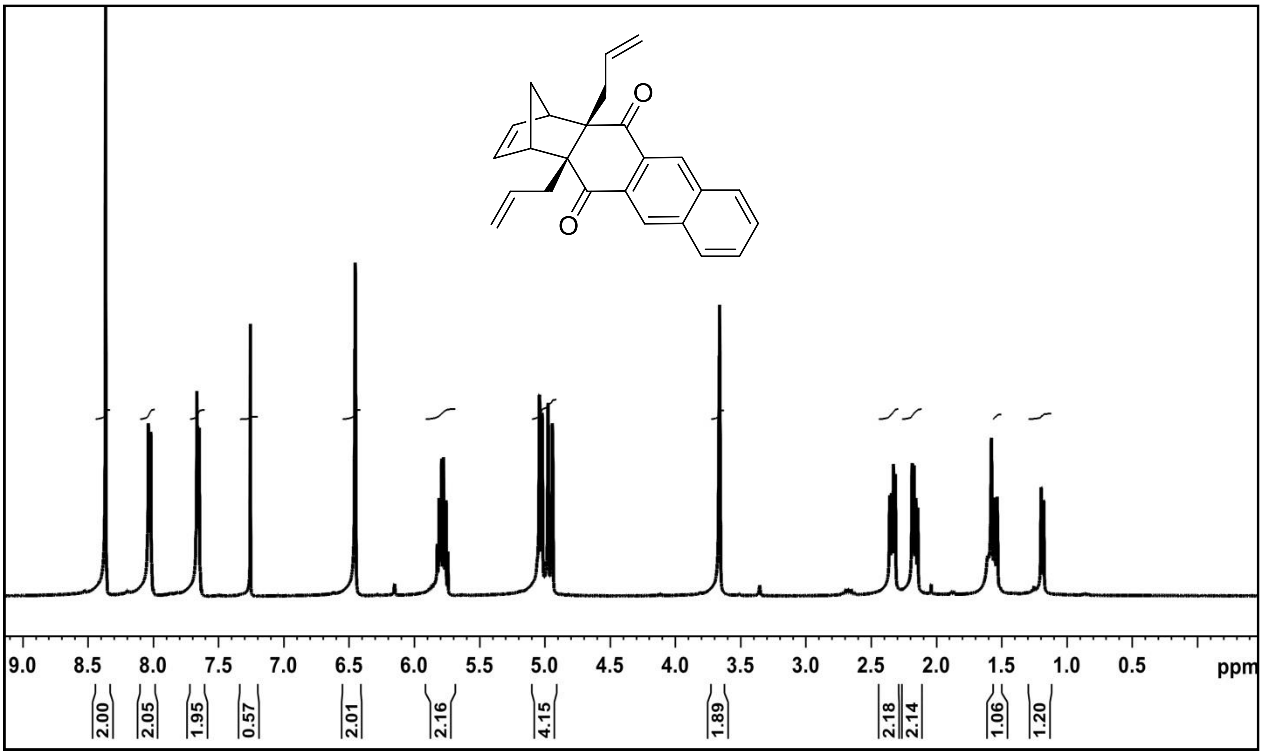

Compound 4b: ^{13}C NMR (100.6 MHz, $CDCl_3$)

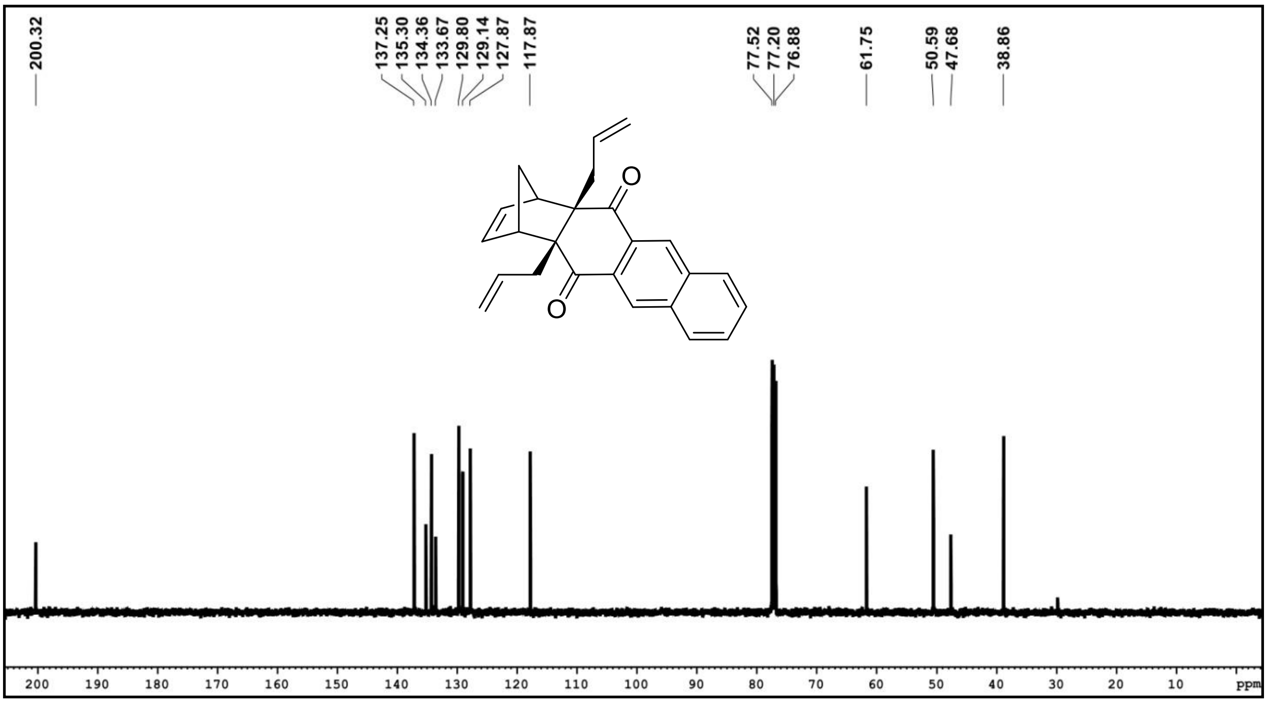

Compound 4b: DEPT-135 NMR (100.6 MHz, $CDCl_3$)

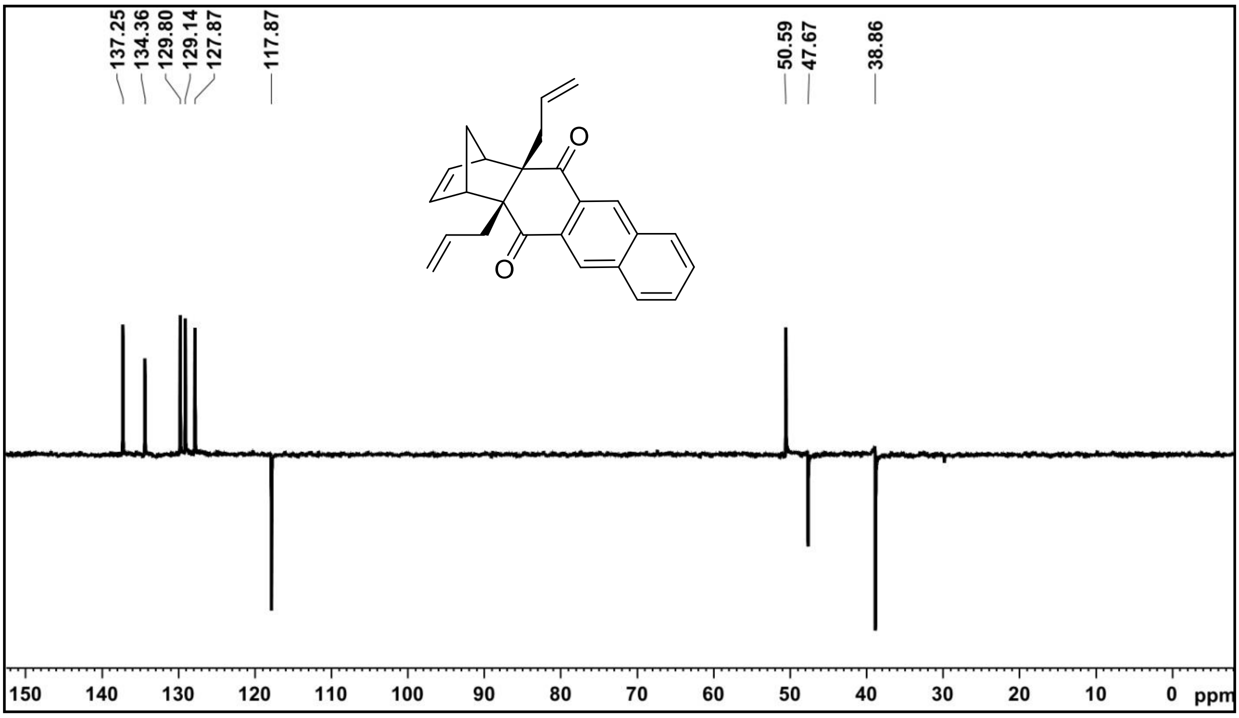

Compound 6a: ^{1}H NMR (400 MHz, $CDCl_3$)

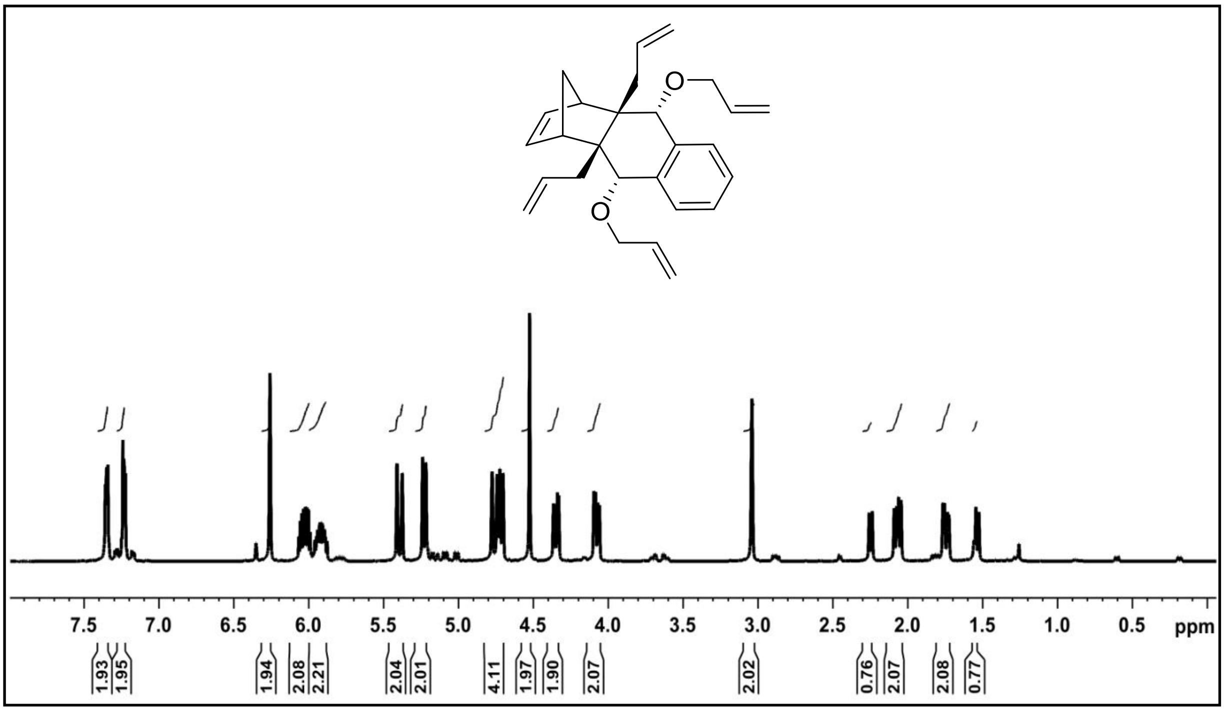

Compound 6a: ^{13}C NMR (100.6 MHz, $CDCl_3$)

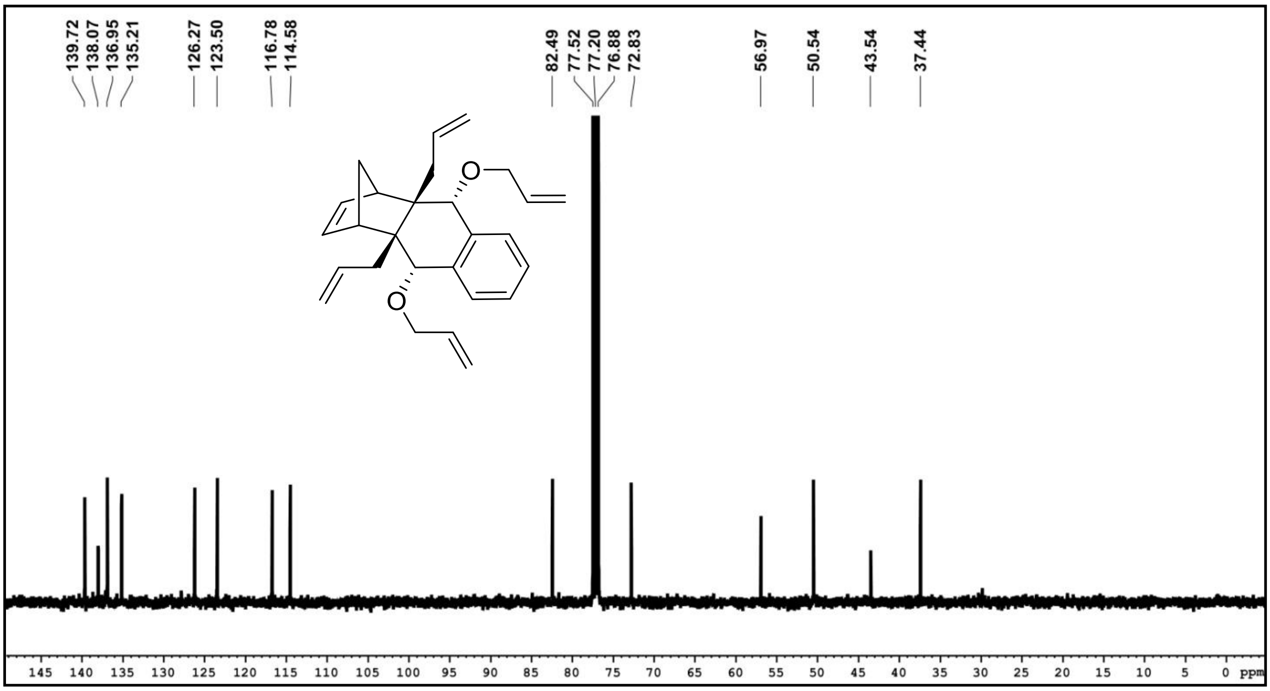

Compound 6a: DEPT-135 NMR (100.6 MHz, $CDCl_3$)

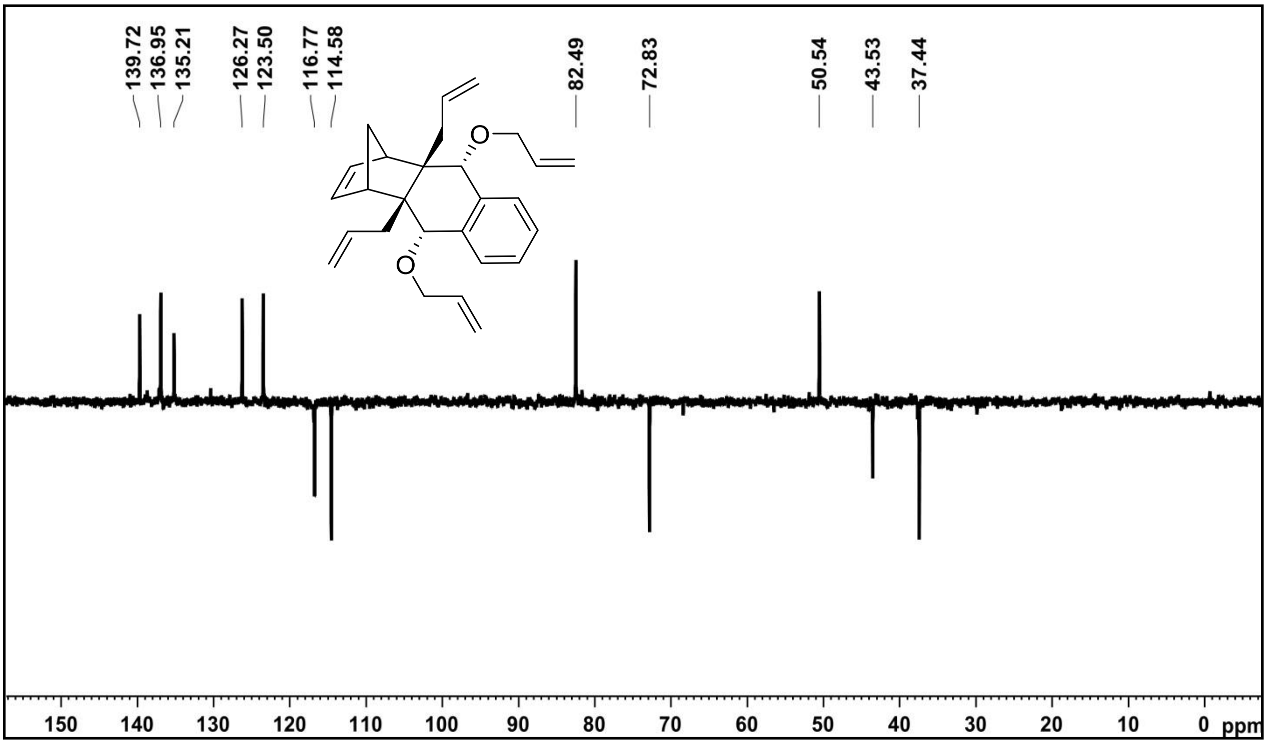

Compound 6b: ^{1}H NMR (400 MHz, $CDCl_3$)

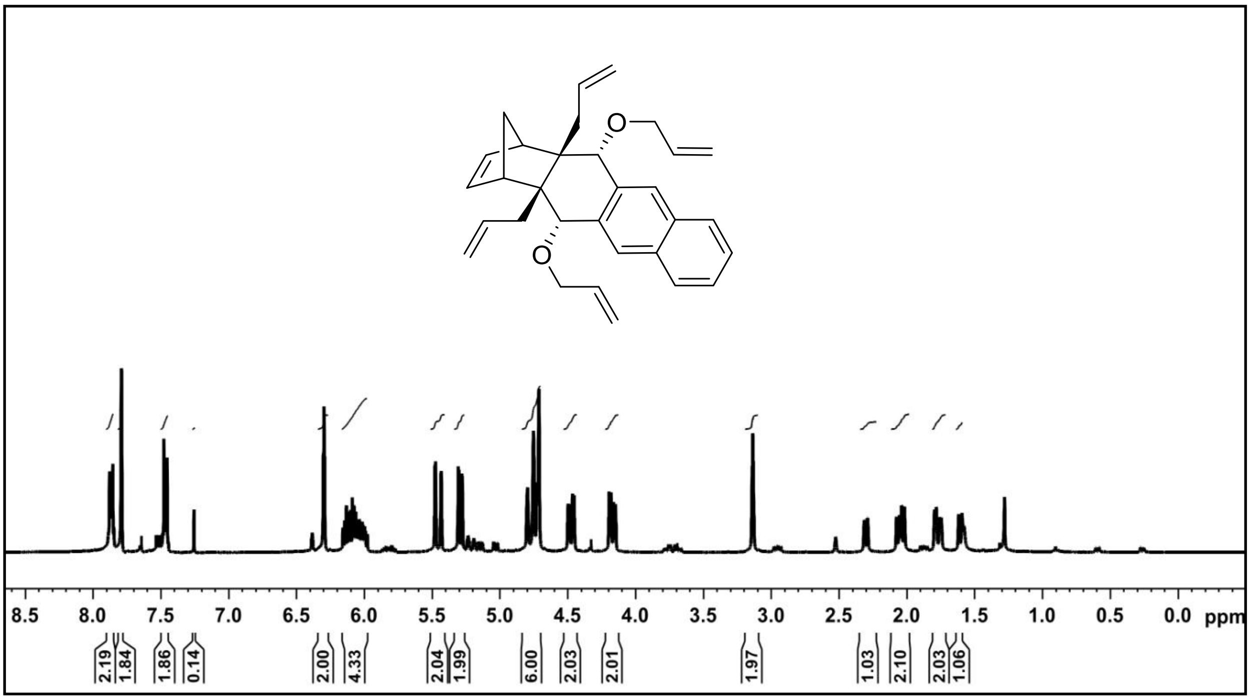

Compound 6b: ^{13}C NMR (100.6 MHz, $CDCl_3$)

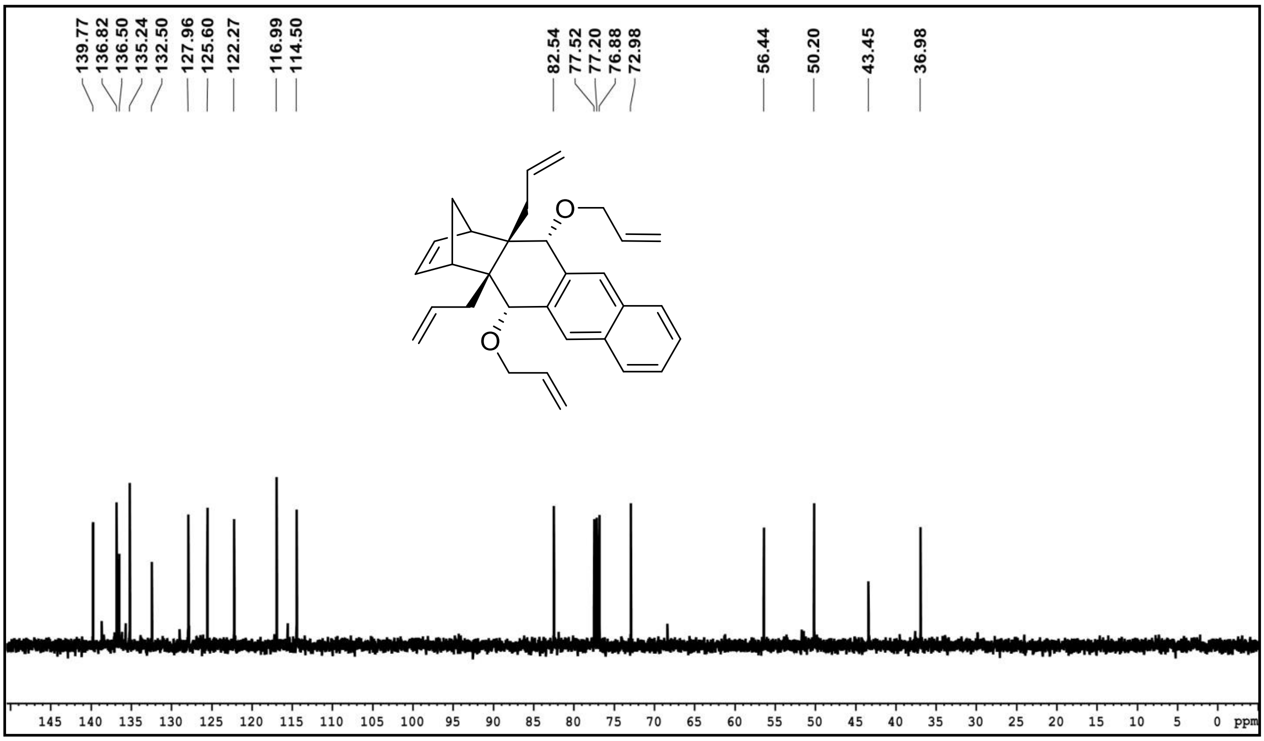

Compound 6b: DEPT-135 NMR (100.6 MHz, $CDCl_3$)

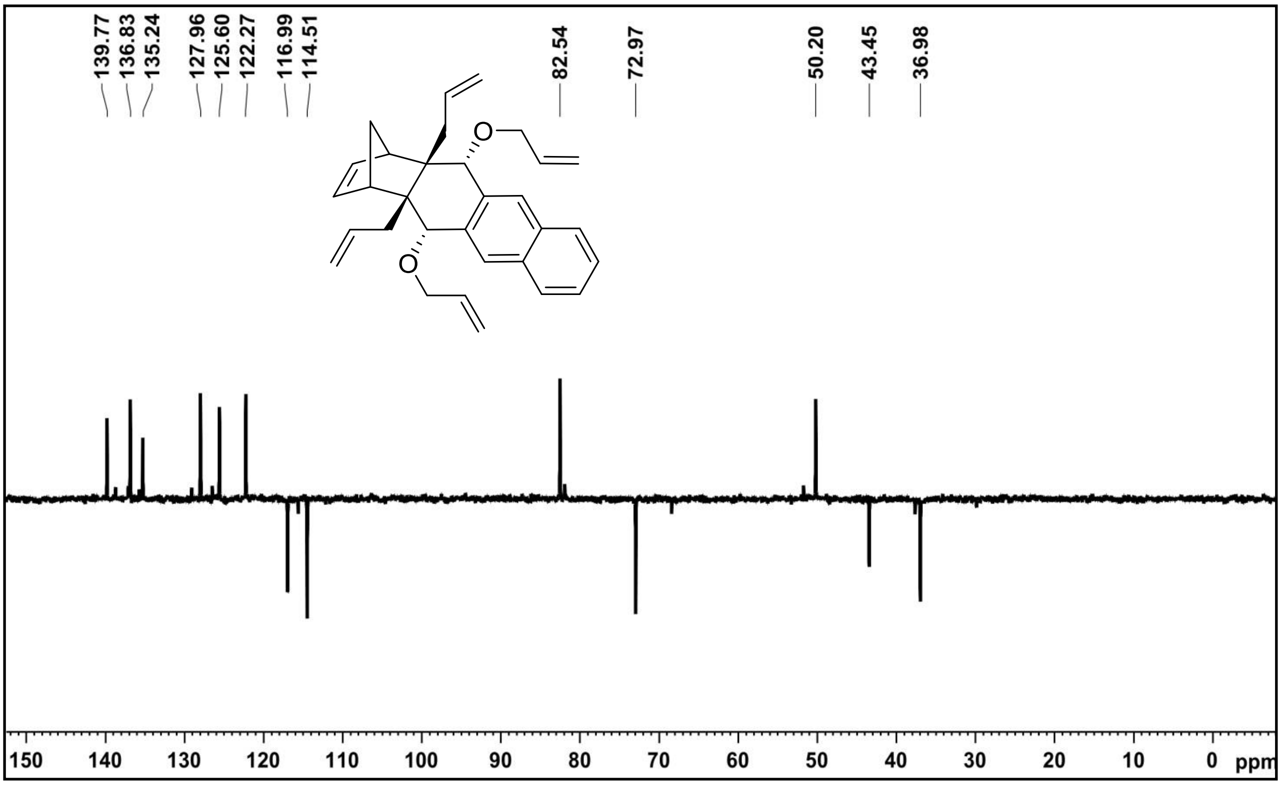

Compound 1a: ^{1}H NMR (400 MHz, $CDCl_3$)

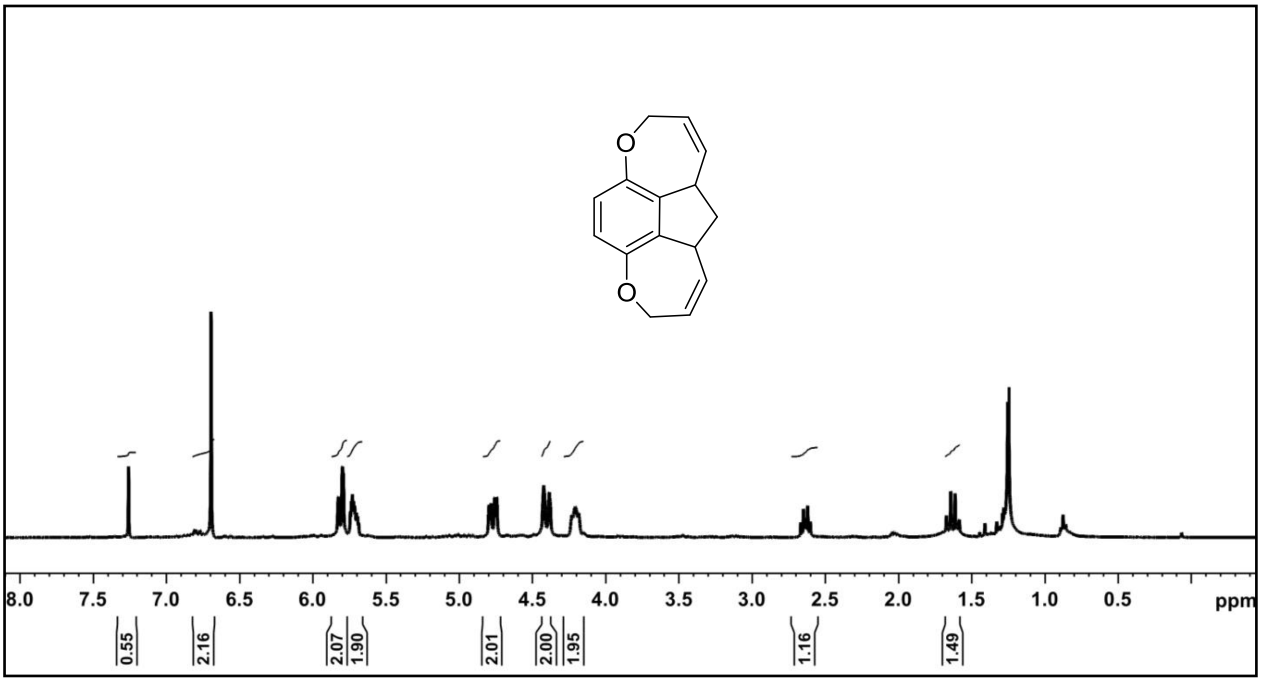

Compound 1a: ^{13}C NMR (100.6 MHz, $CDCl_3$)

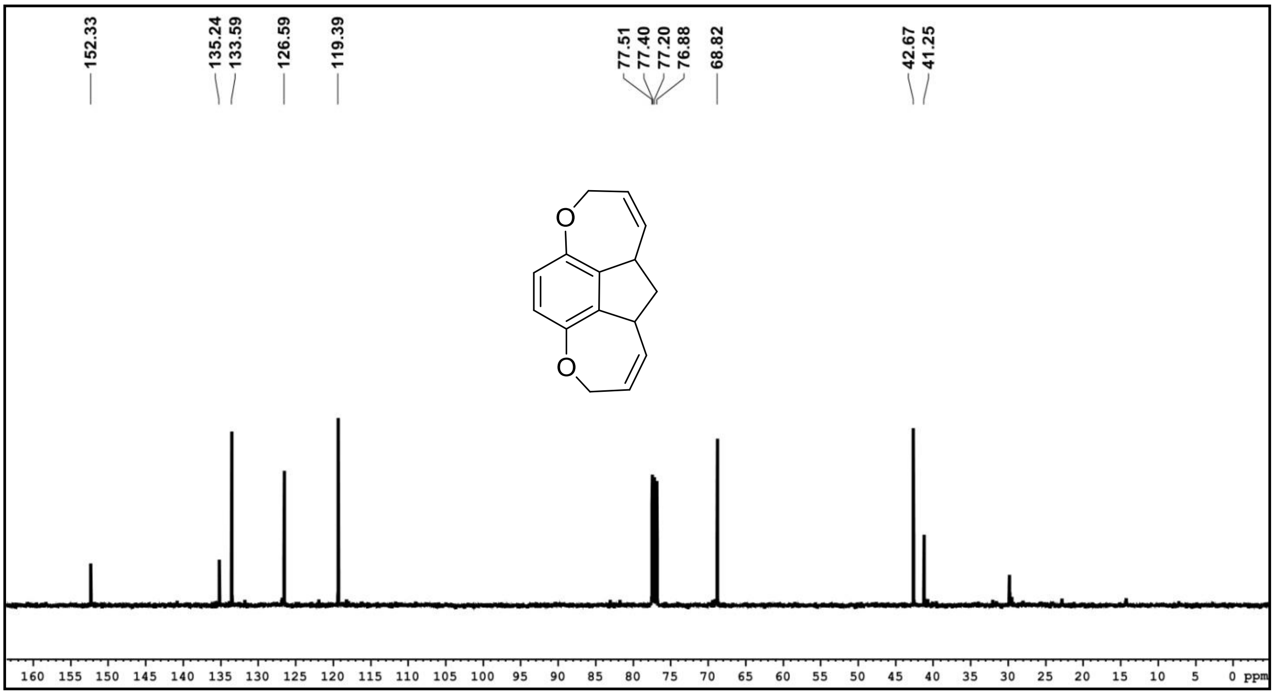

Compound 1a: DEPT-135 NMR (100.6 MHz, $CDCl_3$)

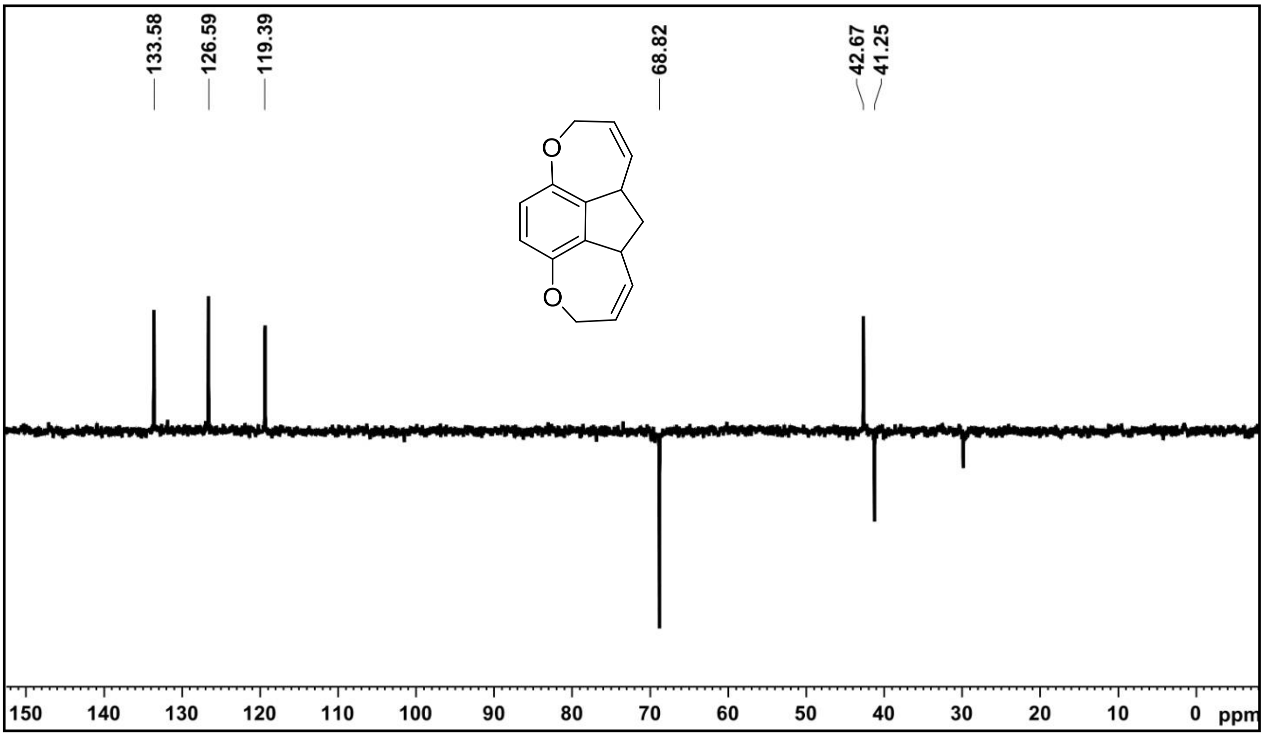

Compound 1b: ^{1}H NMR (400 MHz, $CDCl_3$)

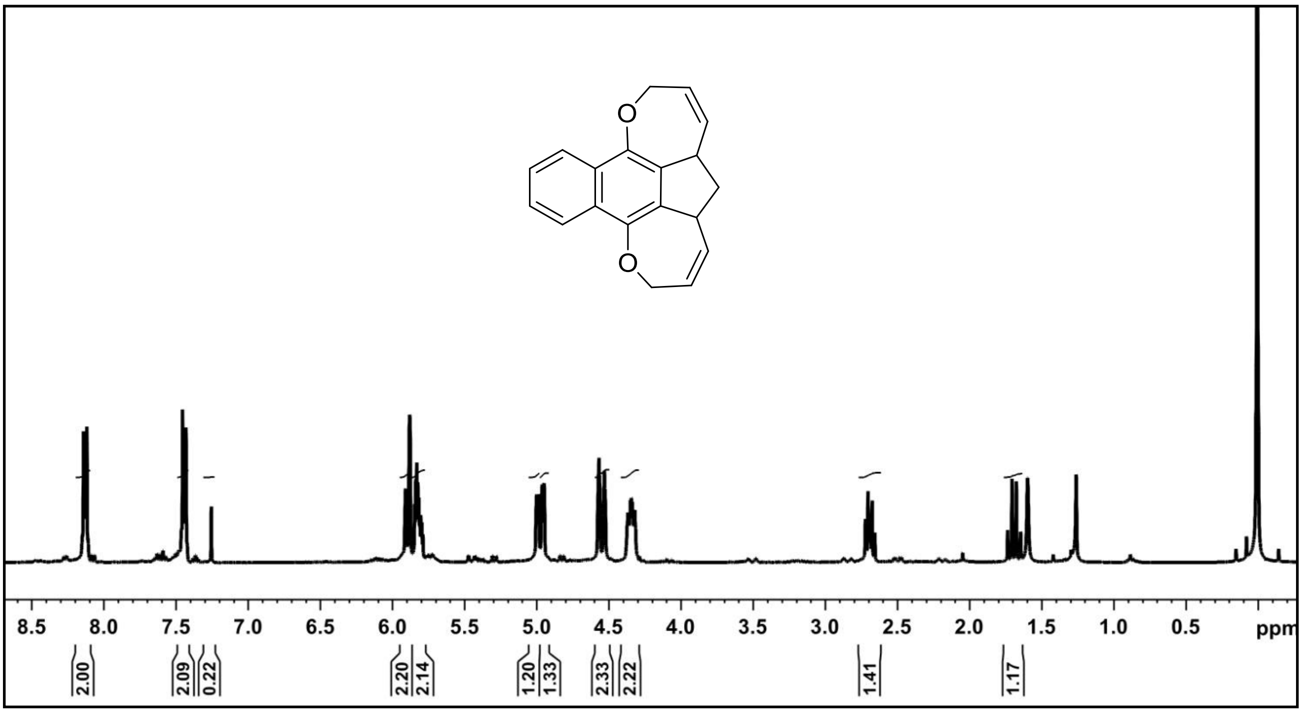

Compound 1b: ^{13}C NMR (100.6 MHz, $CDCl_3$)

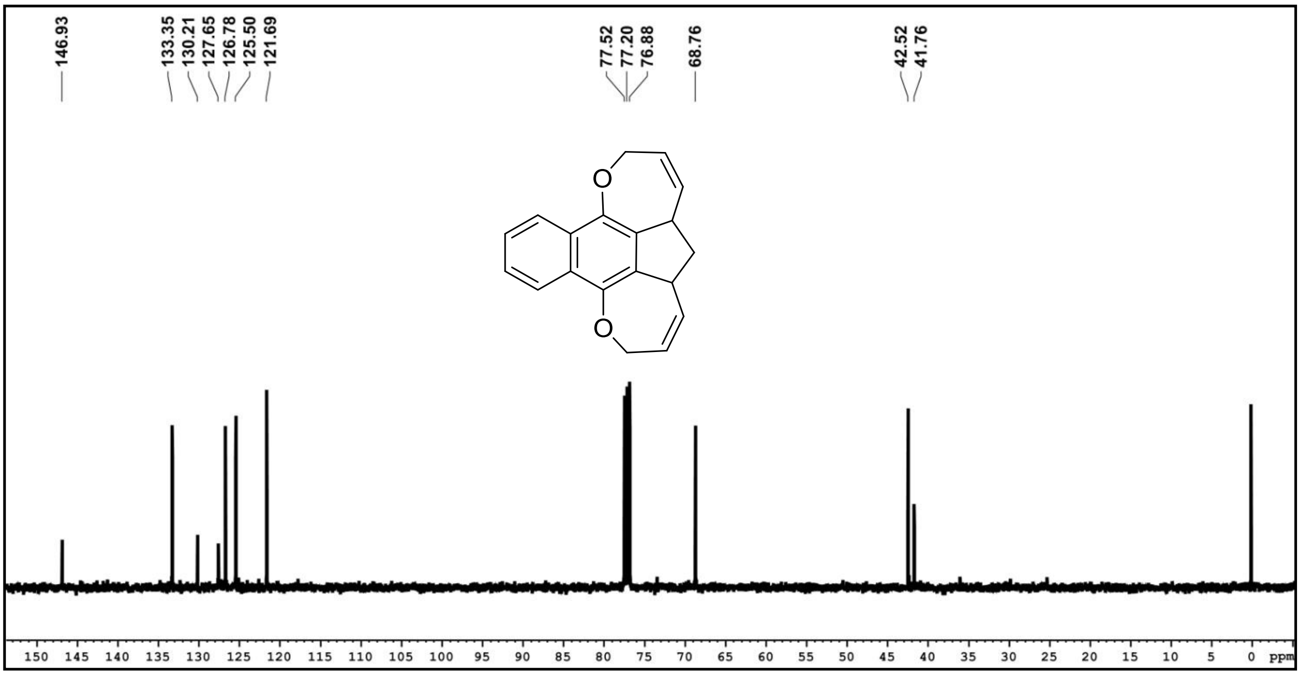

Compound 1c: ^{1}H NMR (400 MHz, $CDCl_3$)

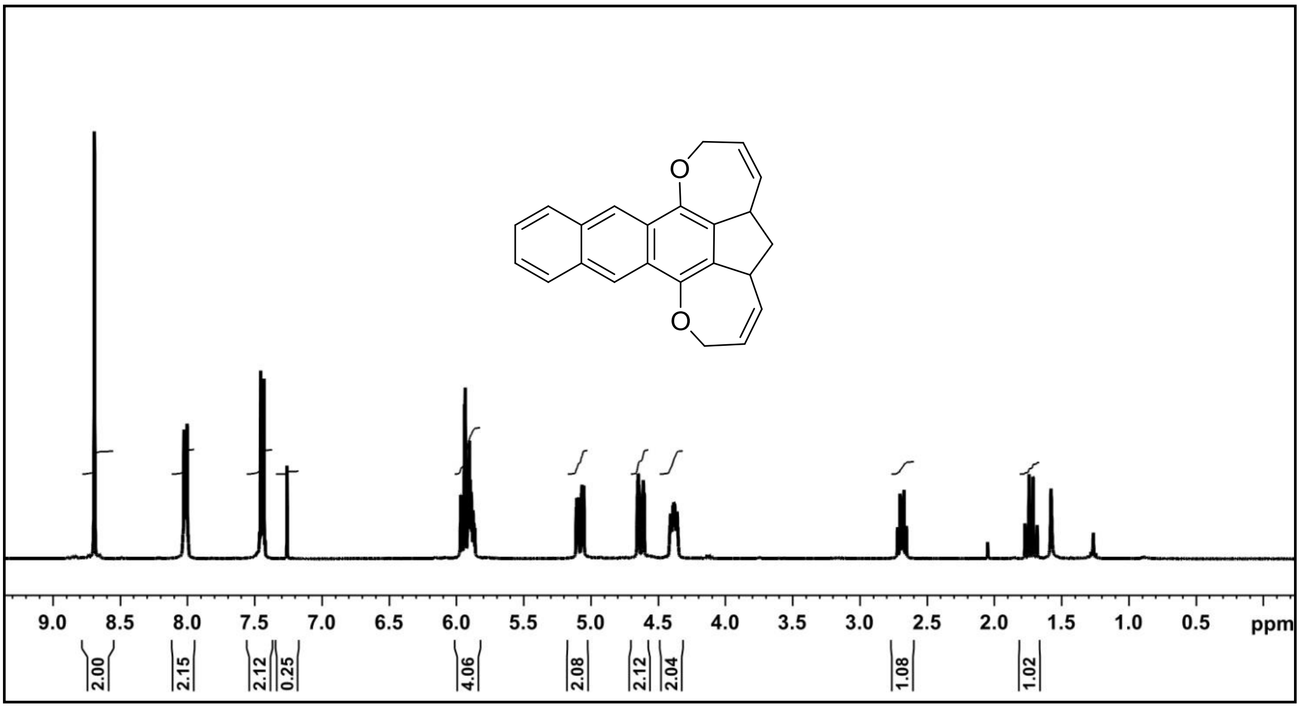

Compound 1c: ^{13}C NMR (100.6 MHz, $CDCl_3$)

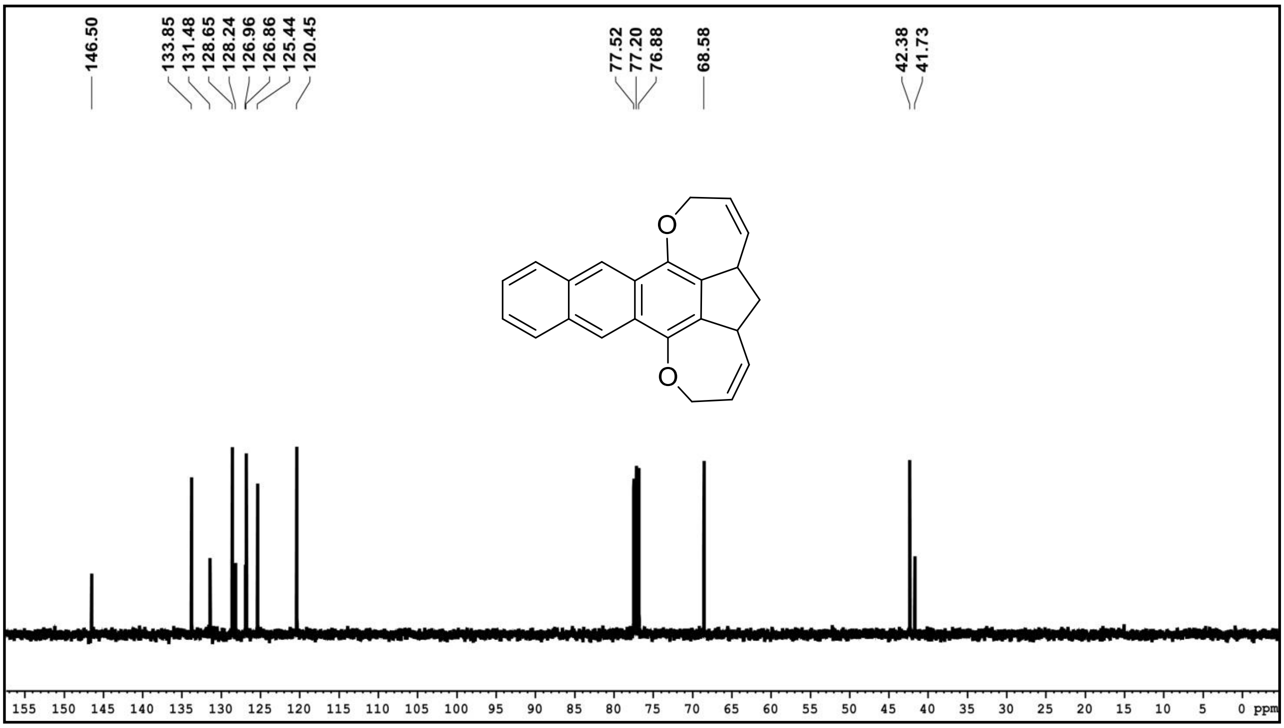

Compound 1c: DEPT-135 NMR (100.6 MHz, $CDCl_3$)

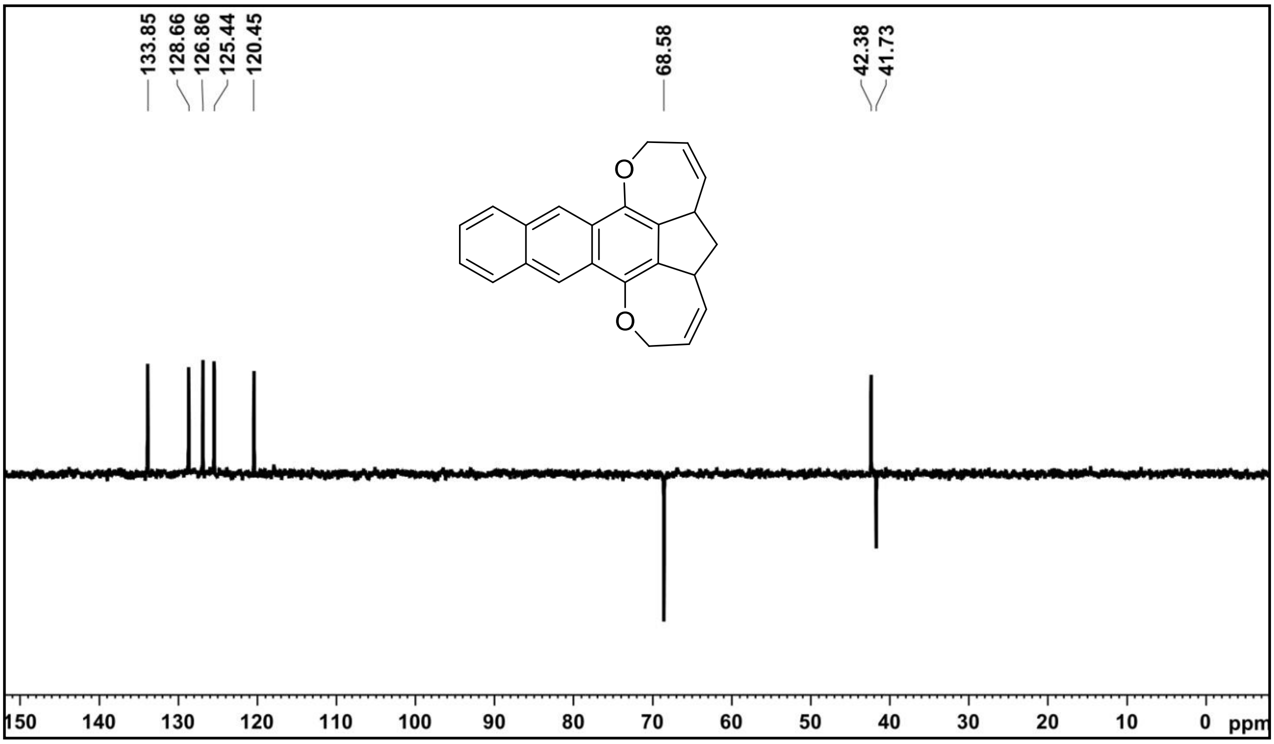

Compound 7a: ^{1}H NMR (400 MHz, $CDCl_3$)

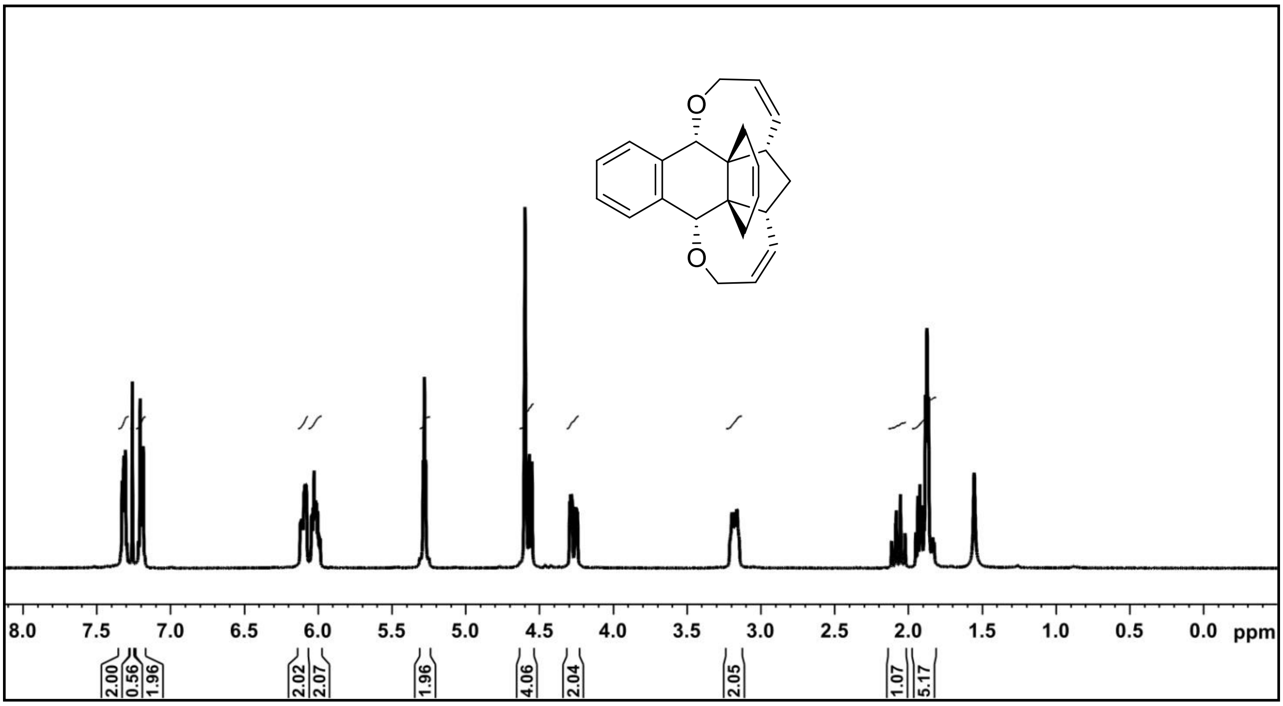

Compound 7a: ^{13}C NMR (125.7 MHz, $CDCl_3$)

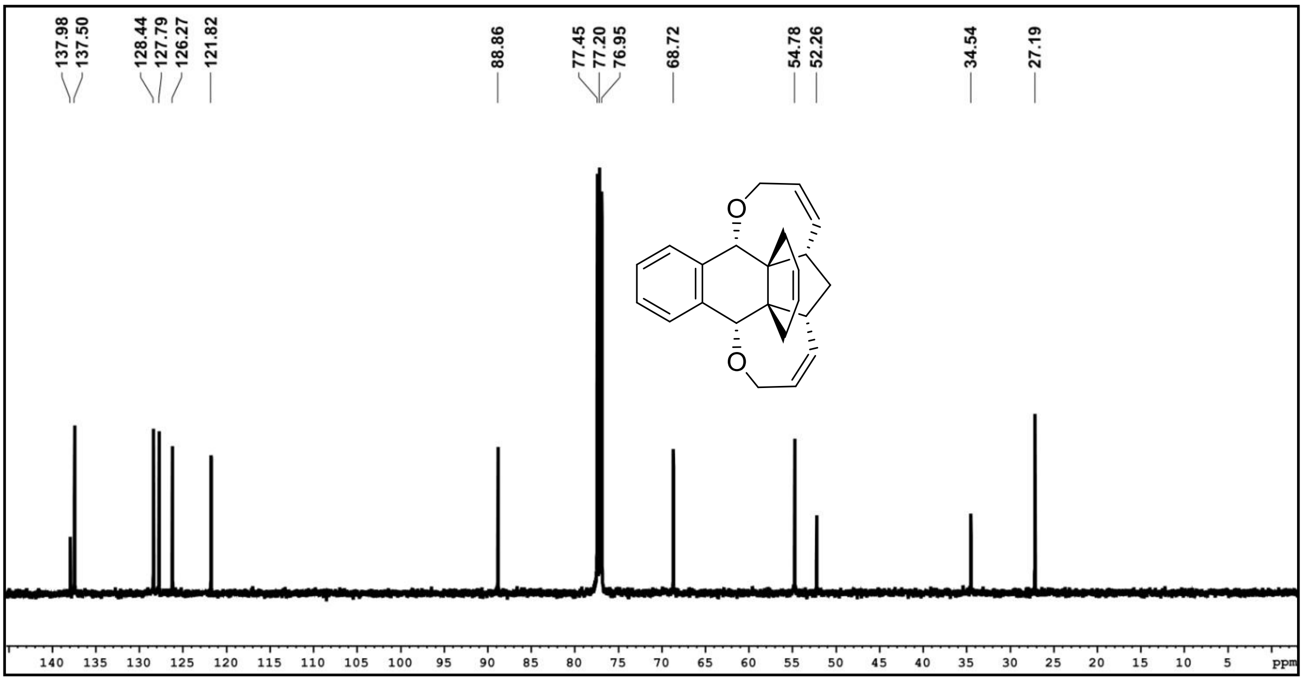

Compound 7a: DEPT-135 NMR (125.7 MHz, $CDCl_3$)

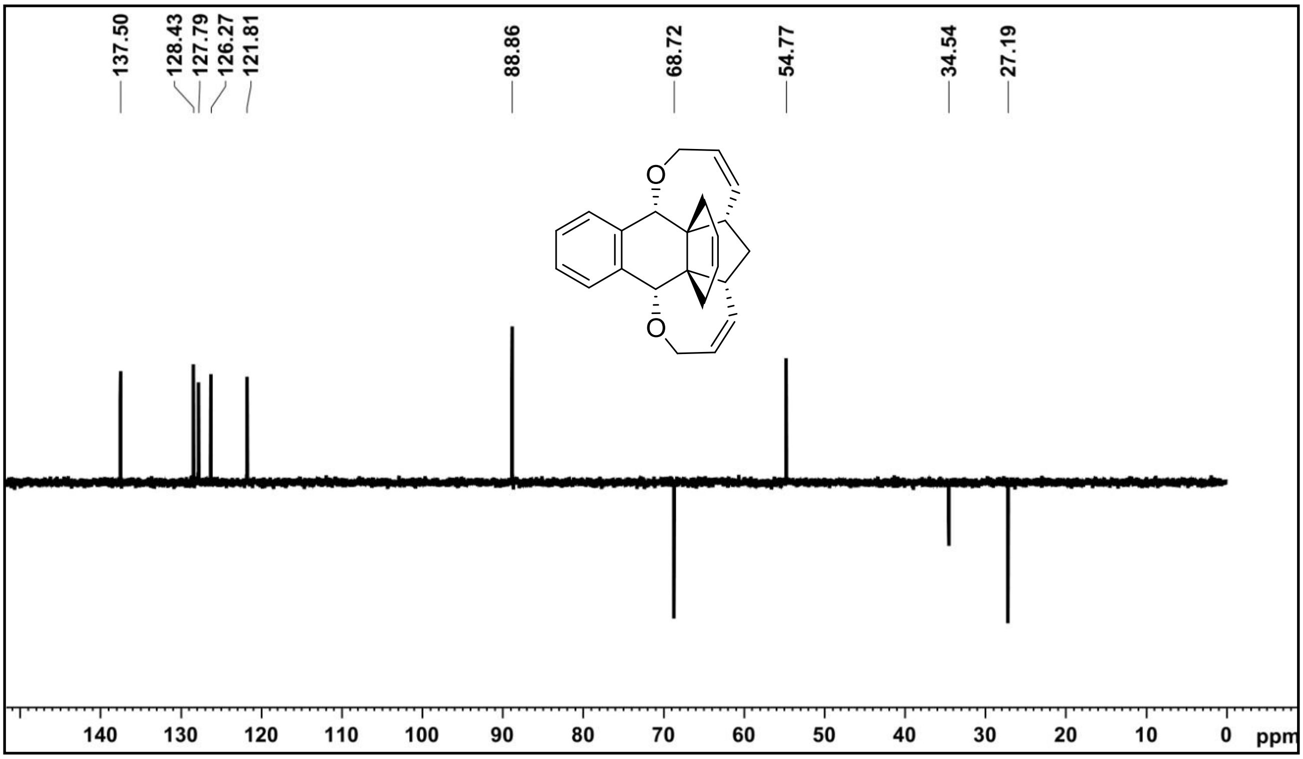

Compound 7b: ^{1}H NMR (400 MHz, $CDCl_3$)

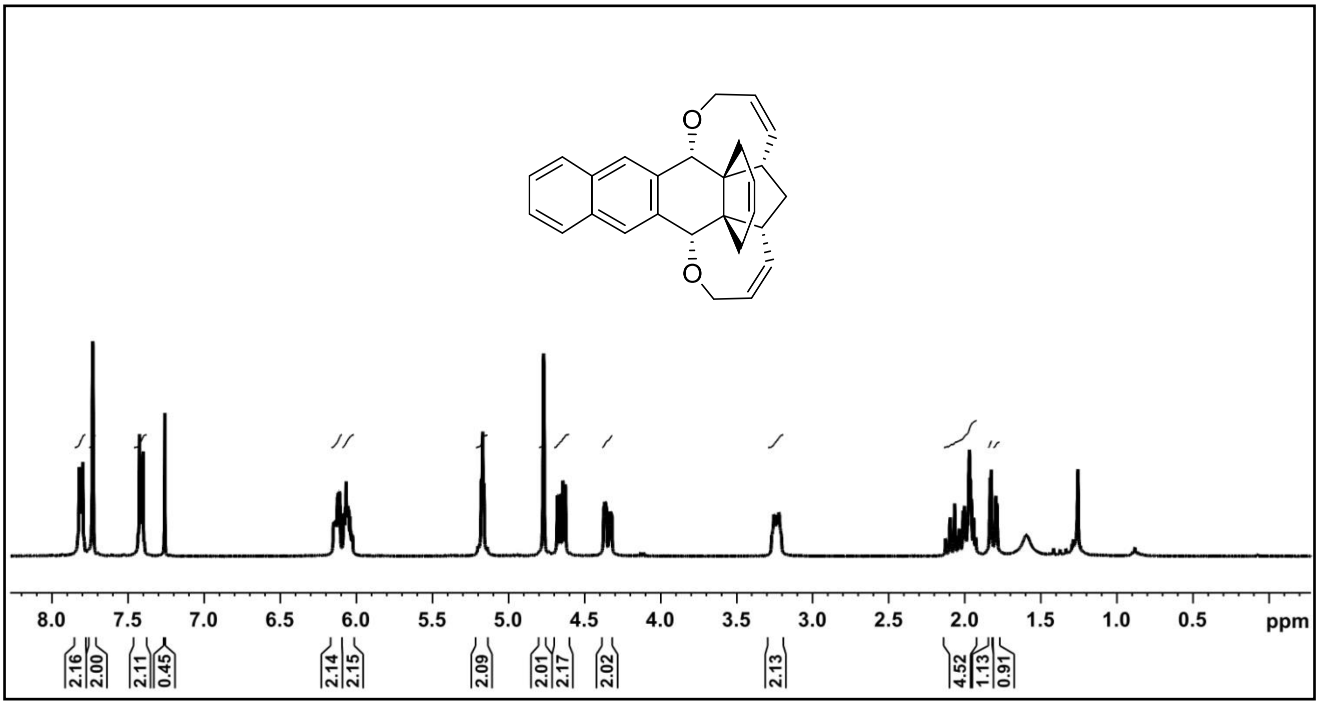

Compound 7b: ^{13}C NMR (100.6 MHz, $CDCl_3$)

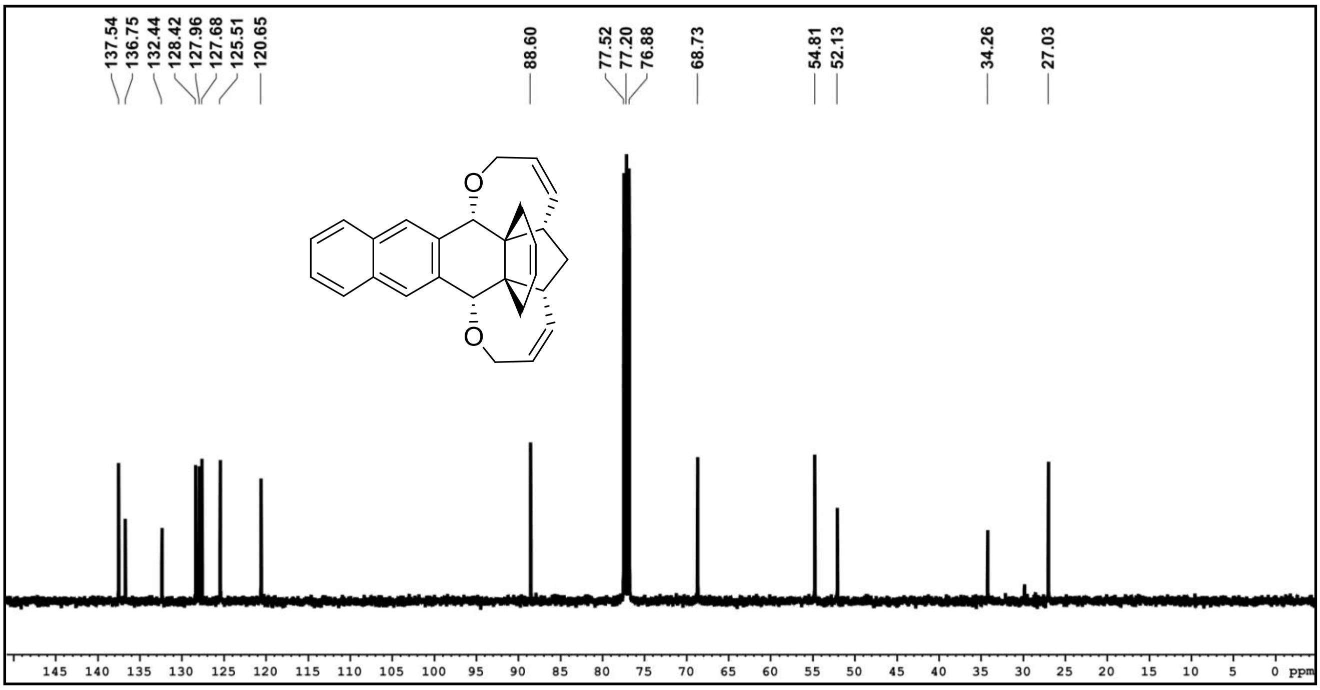

Compound 7b: DEPT-135 NMR (100.6 MHz, $CDCl_3$)

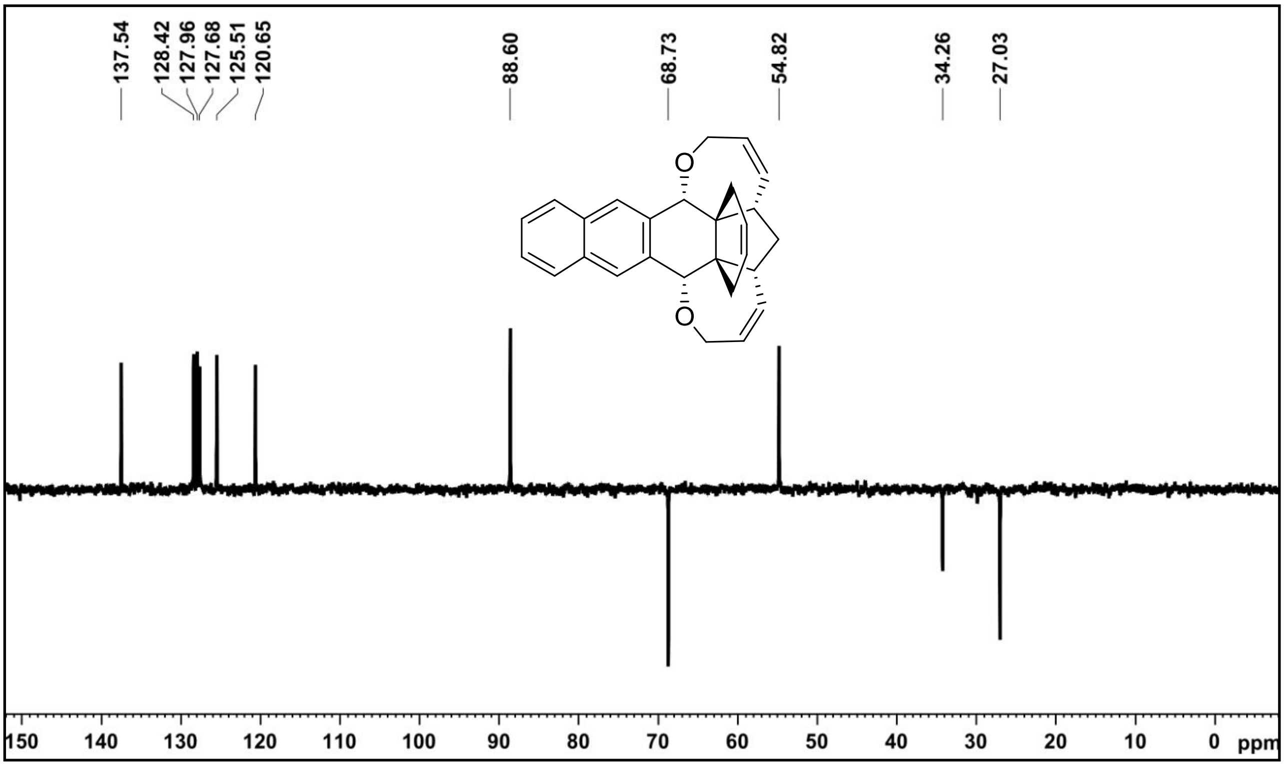

Compound 5a: ^{1}H NMR (400 MHz, $CDCl_3$)

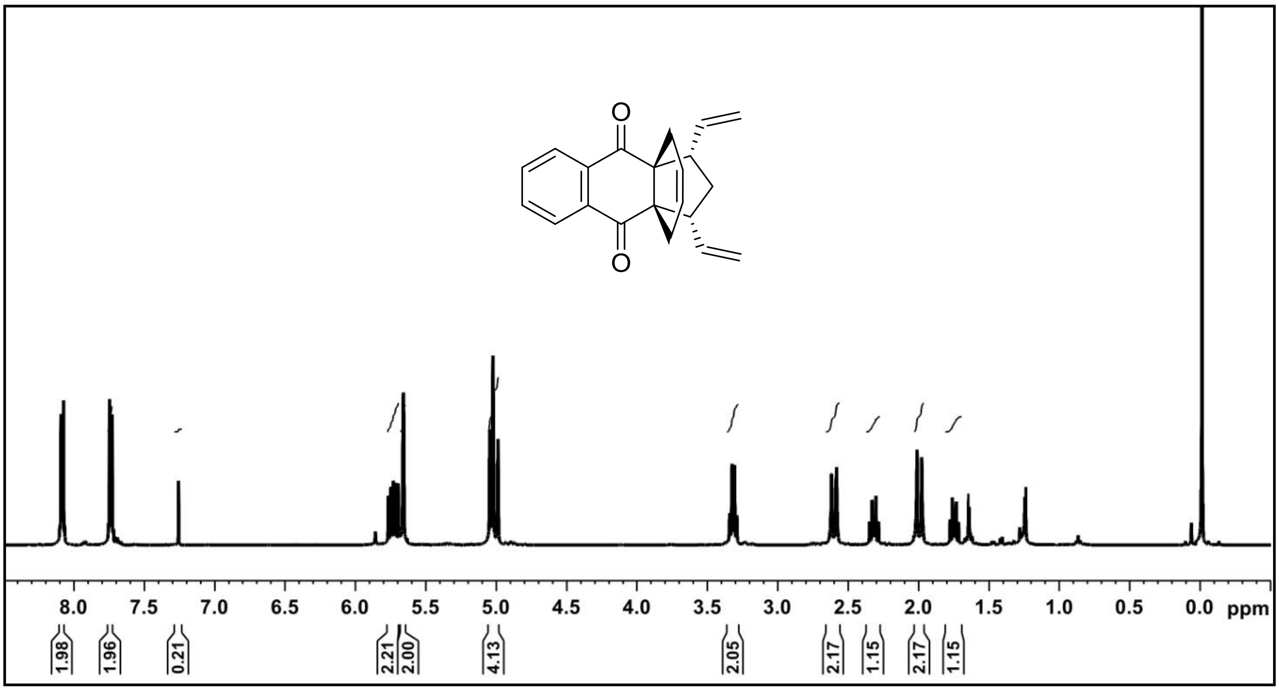

Compound 5a: ^{13}C NMR (100.6 MHz, $CDCl_3$)

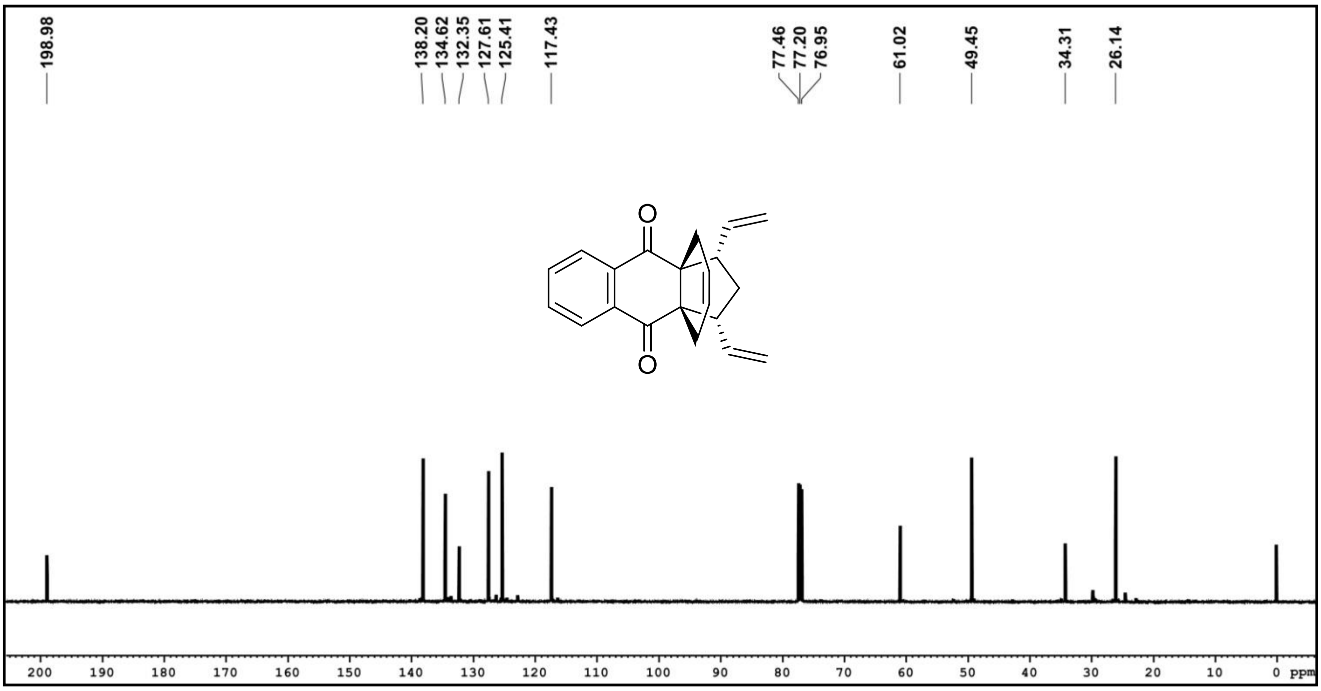

Compound 8a: ^{1}H NMR (400 MHz, $CDCl_3$)

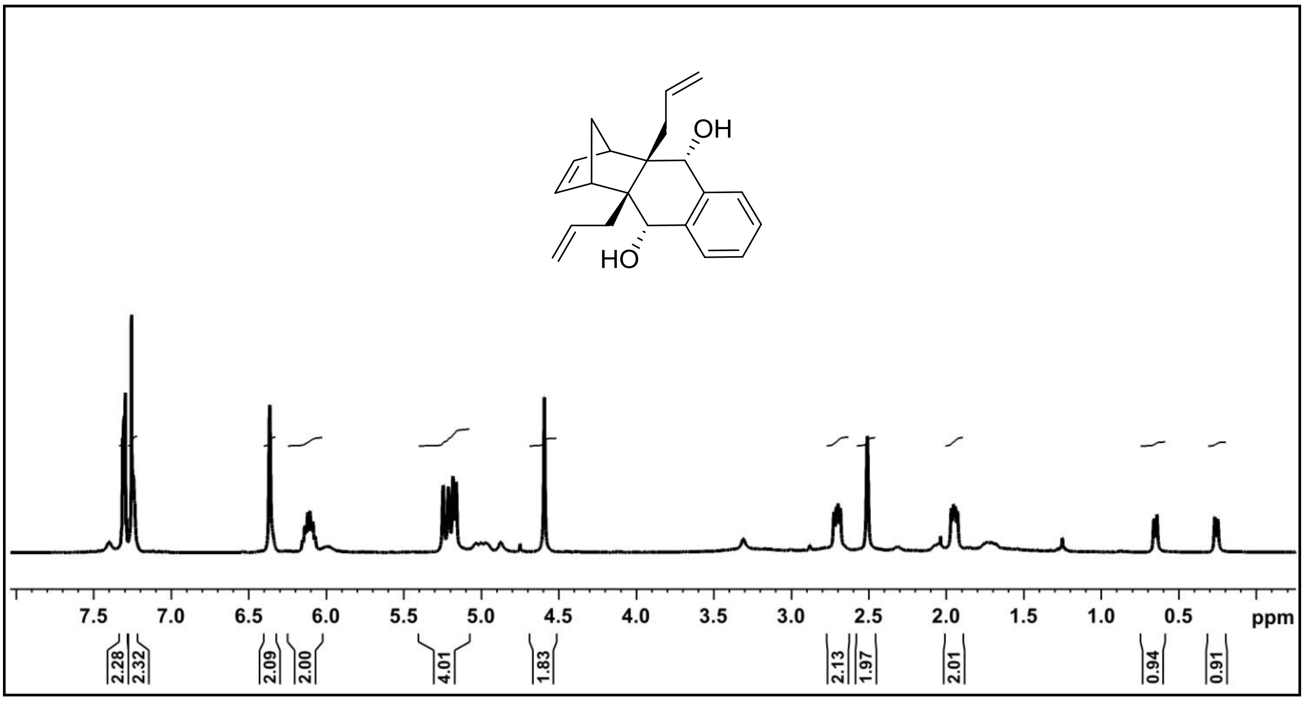

Compound 8a: ^{13}C NMR (125.7 MHz, $CDCl_3$)

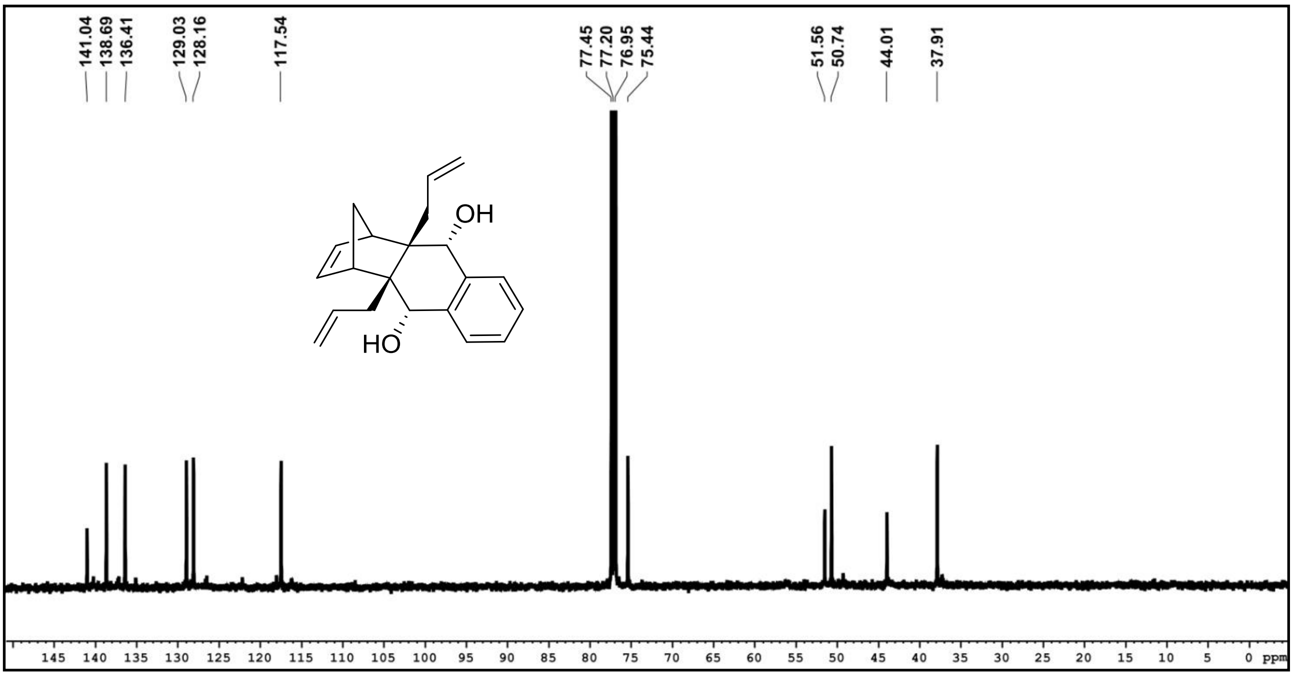

Compound 8a: DEPT-135 NMR (125.7 MHz, $CDCl_3$)

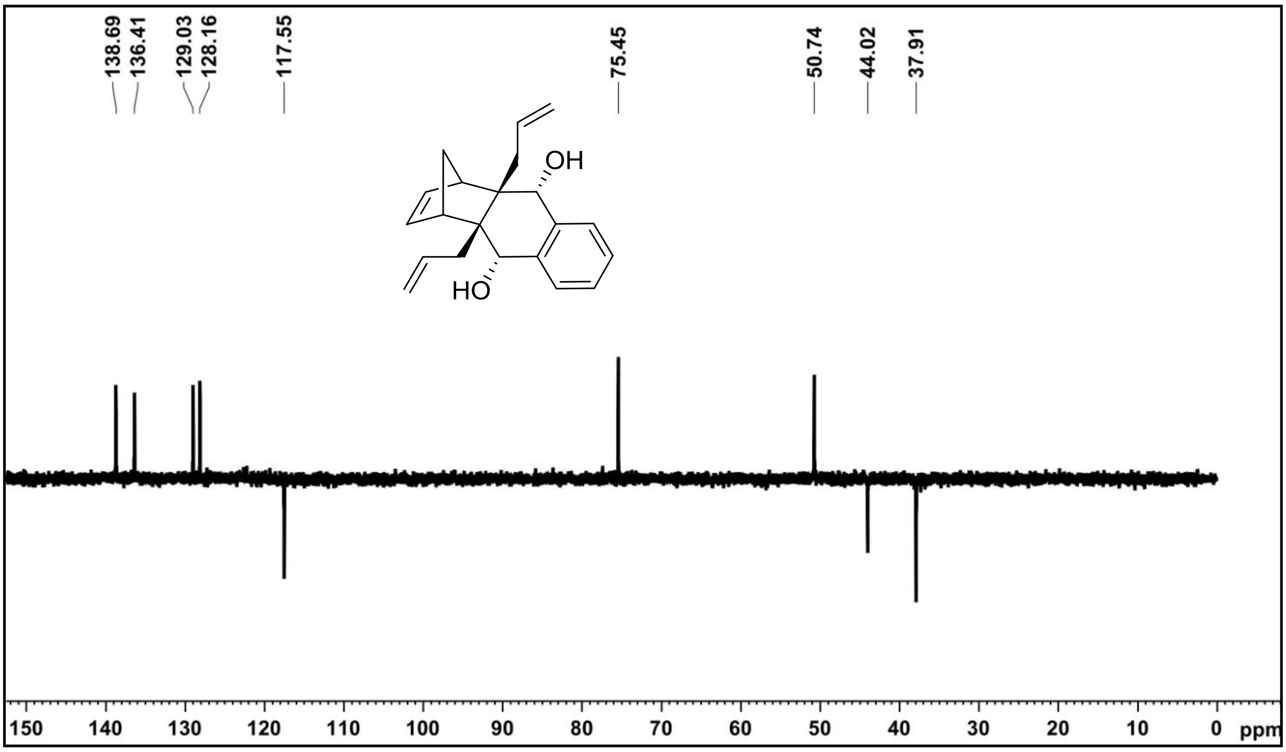

Compound 9: ^{1}H NMR (400 MHz, $CDCl_3$)

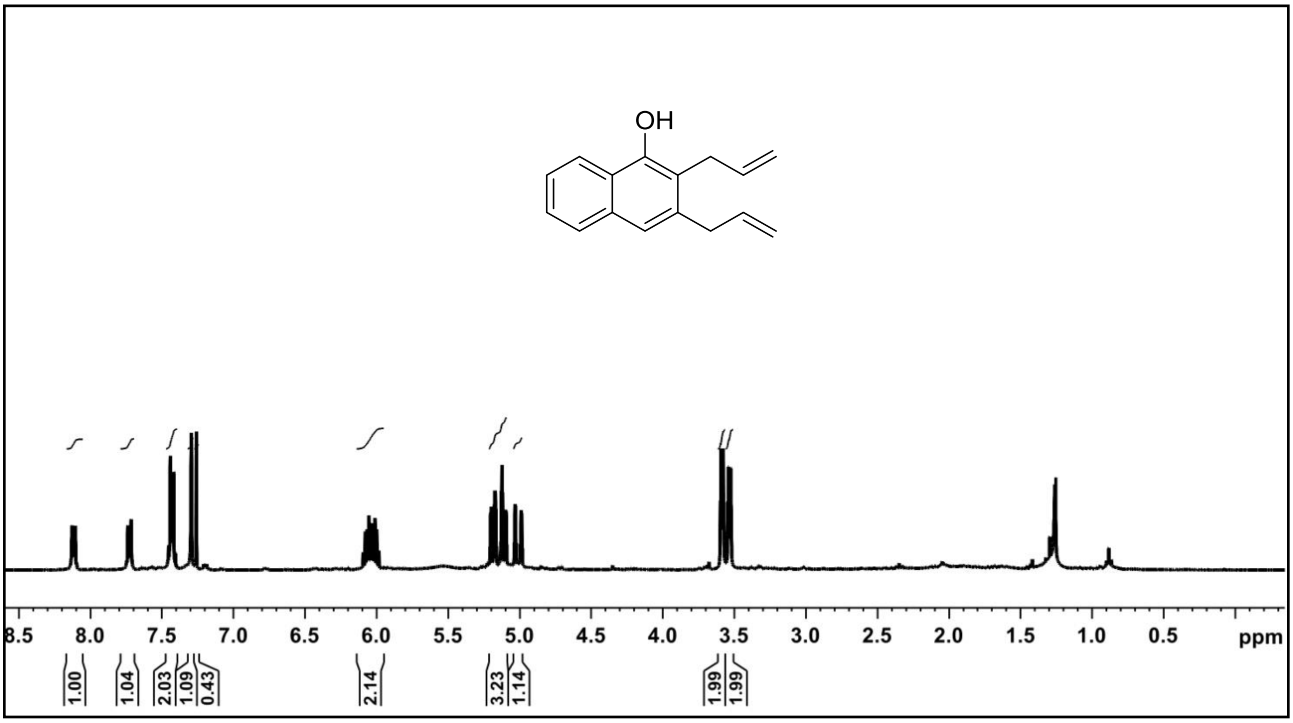

Compound 9: ^{13}C NMR (100.6 MHz, $CDCl_3$)

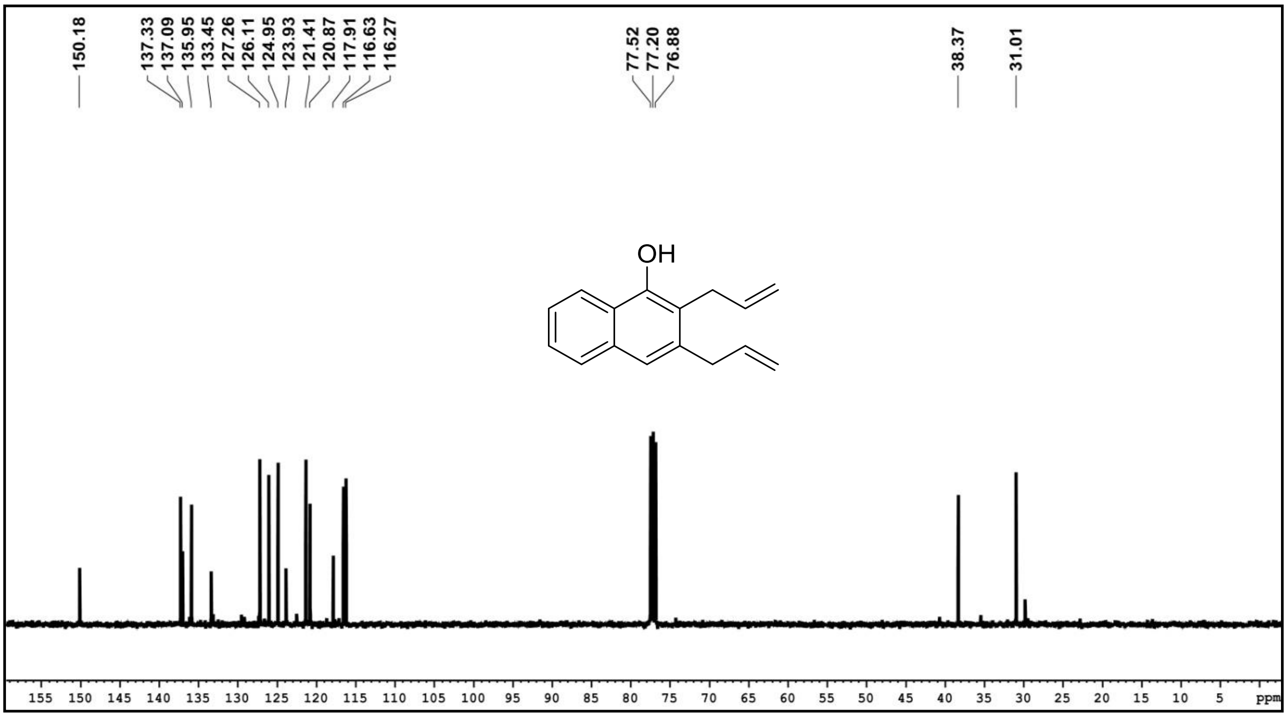

Compound 9: DEPT-135 NMR (100.6 MHz, $CDCl_3$)

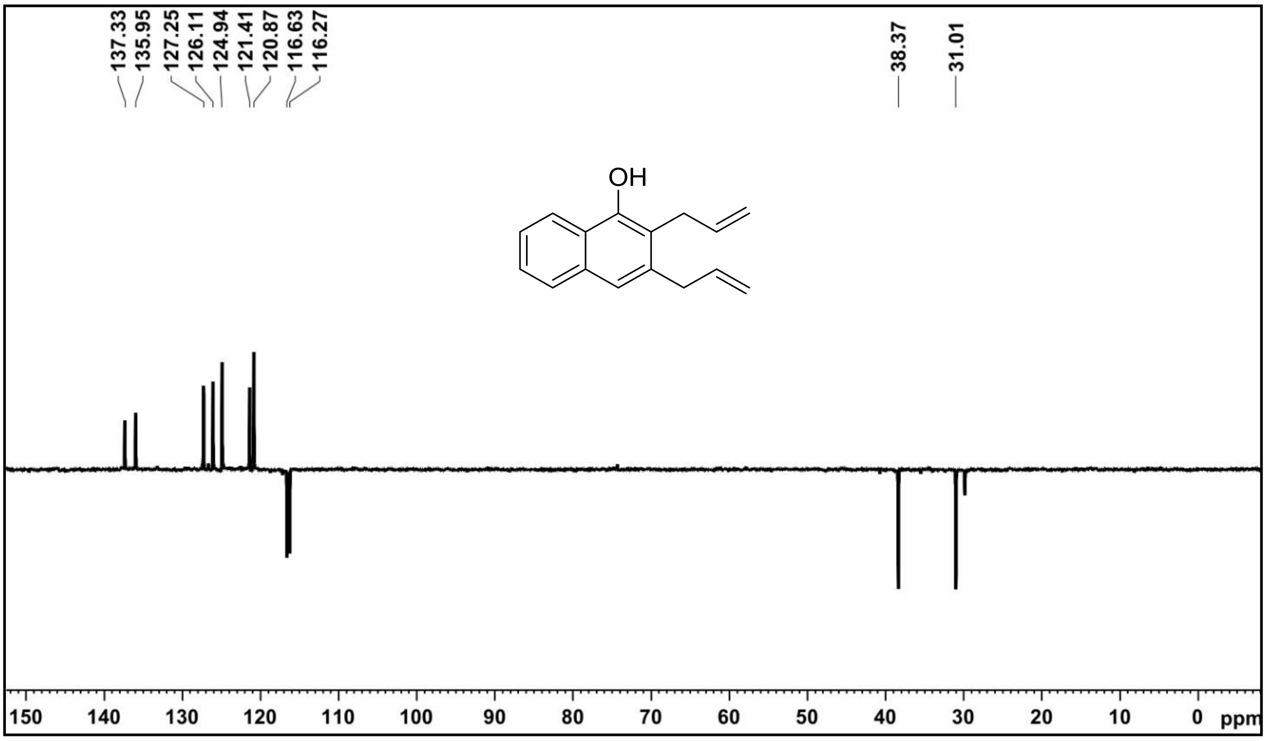

Compound 8b: ^{1}H NMR (400 MHz, $CDCl_3$)

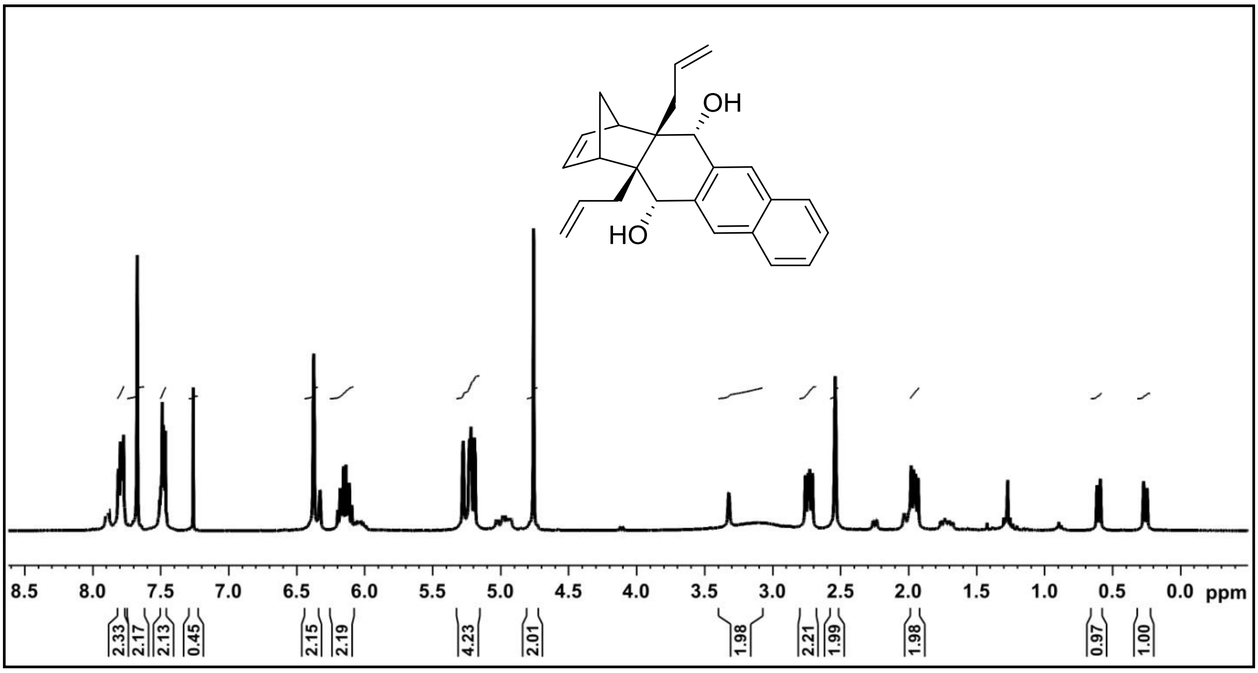

Compound 8b: ^{13}C NMR (100.6 MHz, $CDCl_3$)

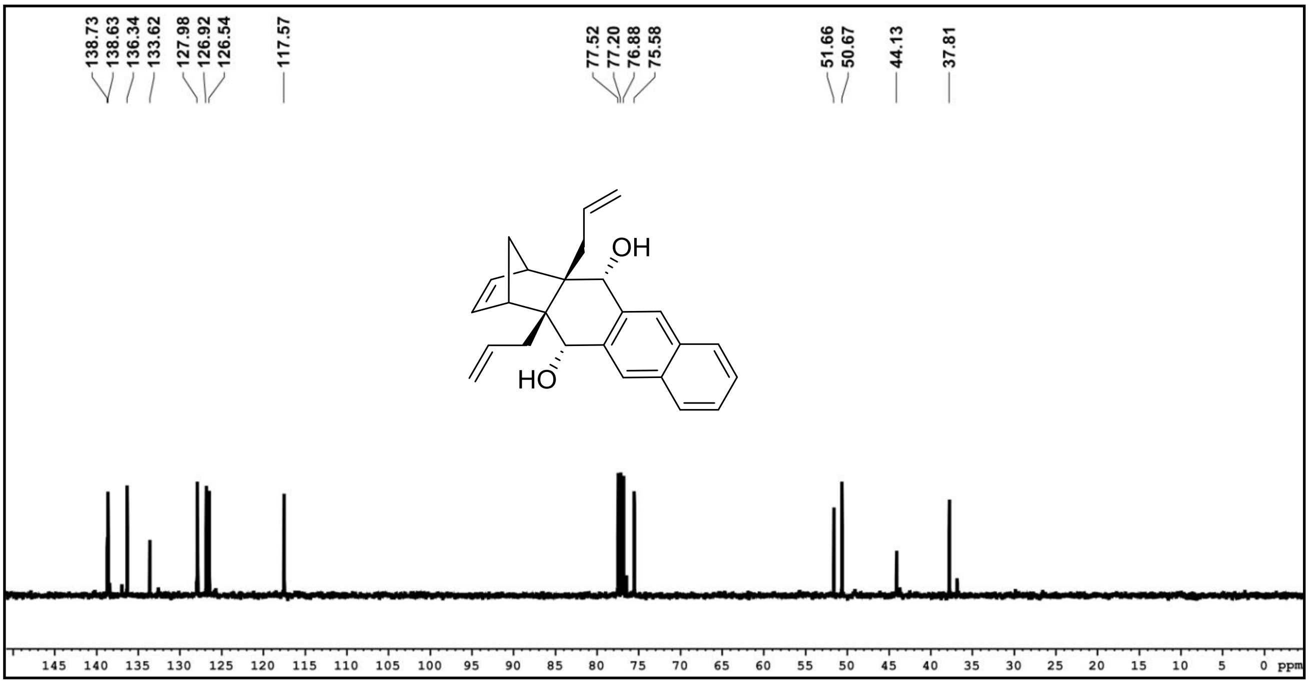

Compound 8b: DEPT-135 NMR (100.6 MHz, $CDCl_3$

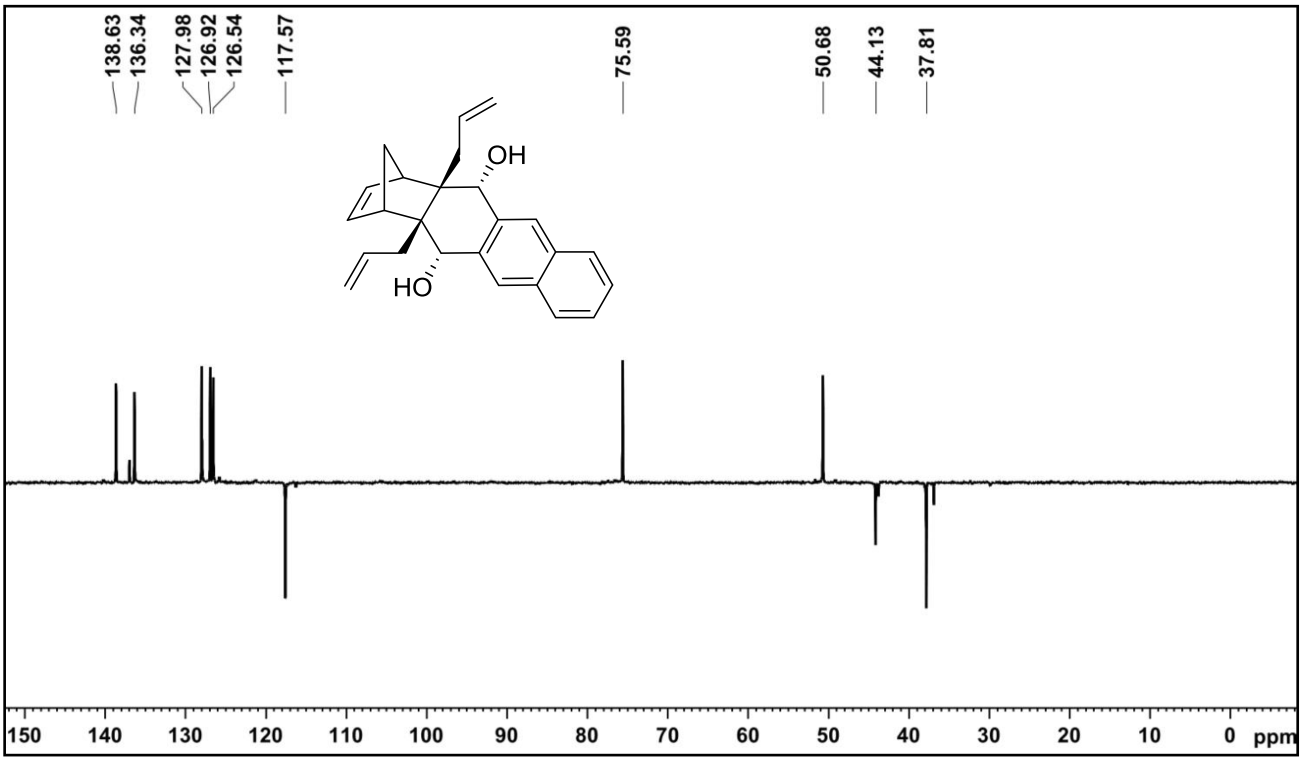

Supplement: File 1 — Detailed experimental procedures, characterization data and copies of 1H and 13C NMR for all new compounds. [file Beilstein_J_Org_Chem-11-1727-s001.pdf]
